# LGR5, a novel functional glioma stem cell marker, promotes EMT by activating the Wnt/β-catenin pathway and predicts poor survival of glioma patients

**DOI:** 10.1186/s13046-018-0864-6

**Published:** 2018-09-12

**Authors:** Jin Zhang, Hongqing Cai, Lixin Sun, Panpan Zhan, Meng Chen, Feng Zhang, Yuliang Ran, Jinghai Wan

**Affiliations:** 1 Department of Neurosurgery, Chinese Academy of Medical Sciences and Peking Union Medical College, National Cancer Center/National Clinical Research Center for Cancer/Cancer Hospital, Beijing, 100021 China; 2 Chinese Academy of Medical Sciences and Peking Union Medical College, National Cancer Center/National Clinical Research Center for Cancer/Cancer Hospital, Beijing, 100021 China

**Keywords:** Glioma stem cell, LGR5, EMT, Wnt/β-catenin, Glioma recurrence, Glioma survival

## Abstract

**Background:**

Tumor recurrence, the chief reason for poor prognosis of glioma, is largely attributed to glioma stem cells (GSCs) and epithelial-mesenchymal transition (EMT). However, the mechanisms among them remain unknown. Here, we determined whether leucine-rich repeat-containing G protein-coupled receptor 5 (LGR5), known as a stem cell marker for colon cancer and gastric cancer, can serve as a novel GSC marker involved in EMT and a therapeutic target in glioma.

**Methods:**

Stemness properties were examined in FACS-isolated LGR5^+^/LGR5^−^ cells. Reported stem cell markers, EMT and the Wnt/β-catenin pathway were examined in stable LGR5 knockdown or overexpressed GSCs by Western Blot. The treatment experiment was performed in an intracranial orthotopic xenograft model by knockdown of LGR5 or by using the Wnt/β-catenin pathway inhibitor Wnt-C59. LGR5 expression was determined in 268 glioma specimens by immunohistochemistry.

**Results:**

LGR5^+^ cells possessed stronger stemness properties compared to LGR5^−^ cells. The expression of SOX2, Nanog, CD133, CD44, CD24 and EpCAM was modulated by LGR5. Both LGR5 knockdown and Wnt-C59 reduced tumor invasion and migration and blocked EMT by inhibiting the Wnt/β-catenin pathway in vitro and suppressed the intracranial orthotopic xenograft growth and prolonged the survival of xenograft mice in vivo. Moreover, LGR5 was positively correlated with Ki67, N-cadherin and WHO grade and negatively correlated with IDH1. Glioma patients with high expression of LGR5 showed significantly poorer prognosis.

**Conclusions:**

LGR5 is a new functional GSC marker and prognostic indicator that can promote EMT by activating the Wnt/β-catenin pathway and would thus be a novel therapeutic target for glioma.

**Electronic supplementary material:**

The online version of this article (10.1186/s13046-018-0864-6) contains supplementary material, which is available to authorized users.

## Background

Glioma is the most common and aggressive primary brain tumor [1]. Depending on the malignancy grade, gliomas can be classified into four levels from I to IV according to World Health Organization (WHO) criteria [2]. Despite multiple rigorous treatments comprising maximal surgical resection, radiotherapy, and chemotherapy, the prognosis remains dismal, with a median overall survival time of approximately 15 months for glioblastoma multiforme (GBM, WHO grade IV) [1, 3]. Epithelial-mesenchymal transition (EMT) is a vital process through which epithelial cells lose cell polarity and adhesiveness and thus transform into mesenchymal cells, which have an increased invasive or metastatic phenotype [4]. This high capacity for invasion and migration caused by EMT often limits total surgical resection and contributes to therapeutic resistance, which eventually leads to tumor recurrence [3–6]. However, the mechanism of signaling pathways and effector molecules that drive glioma EMT and invasion remains not well knowable [4]. Recent developments in stem cell research have revealed the existence of glioma stem cells (GSCs) which are analogous to cancer stem cells (CSCs) and determined that GSCs represent a subpopulation of cells with strong tumorigenesis, invasiveness, chemoradiotherapy resistance and EMT properties [7, 8]. Precisely because of these characteristics, GSCs are considered as responsible for failure of treatment and tumor recurrence [8–12]. Therefore, GSCs are regarded as a relevant target for glioma therapy.

Accumulated evidences have suggested that induction of EMT induces CSC phenotype in tumor cells [13, 14] or human mammary epithelial cells and gives rise to invasive and metastatic CSCs [15, 16]. These findings indicated that the mechanisms for regulating EMT and stemness may be closely integrated [17–19]. However, the underlying mechanisms between GSCs and EMT are not fully understood. Therefore, searching for the association between GSCs and EMT may have significant implications for the exploration of both the causes of glioma recurrence and subsequent therapeutic targets.

The therapeutic strategy targeting CSCs is mainly focused on the direct elimination of CSCs by targeting cell surface markers or specific pathways that are essential for maintaining stemness properties [10, 20]. Although some of CSC markers cluster of differentiation (CD) 133, CD44, CD24, CD90 and epithelial cell adhesion molecule (EpCAM), have been employed to identify in GSCs [10, 21, 22], their functionalities remain defective [20, 23, 24]. Moreover, few such surface markers have been confirmed to be functional in GSCs. Leucine-rich repeat-containing G protein-coupled receptor 5 (LGR5), a seven-transmembrane receptor of the G protein-coupled receptor family related to the Wnt pathway, was initially identified as a marker of intestinal stem cells [25]. LGR5 has been reported as a stem cell marker in several tumors [26–29]. Studies have shown that LGR5 can promote tumor initiation, proliferation and invasion [26, 29–32]. Nakata et al. demonstrated that LGR5 level is positively correlated with pathologic grade and an adverse outcome in glioma [33]. However, the role of LGR5 in GSCs has not yet been determined. Moreover, the relevance of LGR5 to invasion and EMT in glioma remains unknown. Hence, we aimed to investigate whether LGR5 is associated with GSC properties and its relationship with EMT and the Wnt pathway in GSCs.

In this study, we determined the stemness properties of LGR5^+^ and LGR5^−^ glioma cells sorted by fluorescence-activated cell sorting (FACS). The relationship between LGR5 and known CSC markers were examined by flow cytometry (FCM) and Western blot. Using a lentiviral vector and Wnt/β-catenin pathway inhibitor Wnt-C59, we explored the association of LGR5 with EMT and Wnt/β-catenin pathway in GSCs in vitro and in vivo. Moreover, the LGR5 expression was determined in human glioma tissues. The role of LGR5 in GSCs was systematically investigated in biological and clinical conditions, which may provide novel insights into the biological progress of GSCs and aid in the development of evaluation of clinical prognosis and targeted GSCs therapy.

## Methods

### Patients

Patients with a history of other malignant tumor and preoperative adjuvant chemotherapy and/or radiotherapy as well a cause of death unrelated to glioma were excluded from the current study. A total of 268 adult glioma patients who had undergone total tumor resection at the Cancer Hospital Chinese Academy of Medical Sciences between January 2012 and December 2016 were included in this retrospective study. The histological pathologies of all patients were confirmed by at least three experienced pathologists and categorized as low-grade (WHO I-II, *n* = 85) or high-grade (WHO III-IV, *n* = 183) according to WHO criteria. The degree of peritumoral edema was retrospectively assessed by T2W imaging from 268 glioma patients and was divided into two groups according to the maximum diameter of the (maximum diameter > 2 cm, severe; maximum diameter ≦ 2 cm, slight/None). Overall survival (OS) and progression-free survival (PFS) were investigated and recorded from the date of the first surgery to the date of death or the endpoint of this study (September 2, 2017). The clinicopathological characteristics of glioma patients are outlined in Additional file 1: Table S1 and Additional file 2: Table S2. This study was approved by the Ethics Committee of Cancer Hospital, Chinese Academy of Medical Sciences. Written informed consent for the use of resected specimens was prospectively obtained from all patients.

### Primary glioma cells

Fresh glioma specimens (shown as glioma by intraoperative frozen pathology and confirmed by postoperative paraffin pathology) were immediately placed in ice-cold DMEM to slow cell metabolism, avoiding excessive damage at room temperature in vitro. Then, the tissues were washed with DMEM, removed from the blood vessels, minced and dissociated into approximately 1 mm pieces by ophthalmic scissors. The samples were then triturated by a homogenizer (Gentle MACS Dissociator) for 10 min; resuspended in DMEM containing 20% FBS, 1% L-glutamine, and 1% penicillin/streptomycin; and passed through a cell strainer. The filtrate containing cells was maintained at a density of 2 × 10^5^ cells/ml in a cell culture incubator with 5% CO_2_ at 37 °C. The entire process was completed in 45 min without trypsin.

### GSCs enriched from glioma cells

Human glioma cell lines (U251, U87MG, A172) were obtained from the Basic Medical Research Institute of the Chinese Academy of Medical Science (October, 2015). Human primary glioma cells (8591, 7112 and LHH) were derived from glioma specimens of the Cancer Hospital, Chinese Academy of Medical Sciences. The main glioma cells used for this study (U251 and 8591) were authenticated by short tandem repeat (STR) profiling (see Additional files 3 and 4).

Before the enrichment experiment, all parent glioma cells were cultured in DMEM (Invitrogen) with 10% FBS (Invitrogen). To obtain enriched cells, the medium of the parent cells was replaced with a serum-free medium containing neurobasal (Invitrogen) supplemented with 40 ng/ml human fibroblast growth factor (hFGF, Peprotech), 40 ng/ml human epidermal growth factor (hEGF, Peprotech) and 2% B27 (Invitrogen) during cell generation. After 7 days, the parent cells and the enriched cells were respectively digested and dissociated as single cells to perform the FCM.

### Flow cytometry for detection and sorting

Parent cells and enriched cells were incubated with LGR5 (#ab75735; Abcam) and an isotype control (#ab171870; Abcam) and subsequently with secondary antibodies (Alexa Fluor 488) for 30 min at room temperature and washed three times in phosphate buffered saline (PBS) after each reaction to detect the proportion of LGR5 expression. Using the method described above, LGR5^+^ and LGR5^−^ glioma cells were sorted from parent cells by FACS.

To explore the proportion of reported stem cell markers in both LGR5^+^ and LGR5^−^ glioma cells, the cells were incubated with APC anti-human CD133 antibody (#130–090-826; Miltenyi), APC anti-human CD44 antibody (#17–0441-82; eBioscience), APC anti-human CD24 antibody (#311118; BioLegend), APC anti-human CD90 antibody (#328114; BioLegend) or APC anti-human EpCAM (#328208; BioLegend) antibody for 30 min at room temperature. APC anti-human IgG (#409306; BioLegend) was used as an isotype control for these markers. All stained cell suspensions were detected or sorted using an AccuriC6 cytometer (BD Biosciences) and analyzed by FlowJo 7.6.1.

### Proliferation, clone formation and drug resistance assay

The sorted LGR5^+^ and LGR5^−^ cells were seeded into 96-well culture plates at a density of 1 × 10^3^ cells per well in 100 μL of medium with 10% FBS. The cells were then cultured for 1–5 days, and the cell numbers were determined by a Cell Counting Kit-8 (CCK8, Dojindo) assay according to the manufacturer’s instructions. Then, 10 μL of CCK8 solution was added to each well of the plate for 1 h of incubation and the absorbance was measured using a microplate reader at 450 nm.

To examine the ability of clone formation, 600 μL of serum-free medium supplemented with 0.8% methyl cellulose, which contained 500 LGR5^+^ or LGR5^−^ cells, was added to each well of the 24-well ultra-low attachment plates (Corning). Every 5 days, we replenished the medium and the colonies were counted after 14 days. The tumor spheres larger than 50 μm in diameter were evaluated in this study.

Temozolomide (TMZ, Sigma) was dissolved in dimethyl sulfoxide (DMSO; Sigma) and a final DMSO concentration was diluted to under 0.5% by 10% FBS DMEM medium. LGR5^+^ and LGR5^−^ cells were cultured with TMZ for 72 h at concentrations of 0, 25, 50, 100, 200, 400 and 800 μM. The absorbance was detected using CCK8 assays by a microplate reader at 550 nm. The IC_50_ was calculated by Graphpad 5.0. Each aforementioned assay was performed in triplicate.

### Lentiviral transfection

All lentiviral vectors for LGR5 knockdown and overexpression were designed and synthesized by Genecopoeia Ltd. (Guangzhou, China), and expressed green fluorescent protein (GFP) and puromycin-resistant proteins for screening. The target sequence of the LGR5 shRNA (shLGR5) was 5′-GCTCTCATCTTGCTCAATTCC-3′, and the target sequence of negative control shRNA (shCtrl) was 5′-GCTTCGCGCCGTAGTCTTA-3′. LGR5 is overexpressed in glioma cells with the transfection of lentivirus containing LGR5 (Lenti-LGR5). Glioma cells infected with lentivirus containing empty vectors were used as negative controls (Lenti-GFP). The transfection process was performed according to the manufacturer’s protocol. The multiplicity of infection (MOI) was 5 for U251 and 10 for 8591. Stably transfected cells were selected by addition of 1 μg/ml of puromycin (Gibco) at 3 days after infection for 1 week. The transfection efficiency was confirmed by FACS and Western blot.

### Invasion and migration assays

The invasion of glioma cells was assessed in transwell chambers (8-μm pore size, Miltenyi) coated with Matrigel (BD Biosciences; 1:4 volume) in accordance with the manufacturer’s protocol. A total of 3 × 10^4^ glioma cells suspended in 100 μL of serum-free DMEM were added onto the upper chamber of a transwell plate, and 600 μL of normal culture medium of 10% FBS was added to the lower chamber. After 24 h of incubation, the cells on the upper surface of the chamber were removed, and the cells on the lower surface were fixed by methanol for 15 min, stained by 1% crystal violet for 30 min and counted. To investigate the effect of Wnt-C59 (APExBio) on the invasion and migration GSCs, Wnt-C59 was completely dissolved in DMSO with a concentration of 2.0 × 10^4^ μM, and the GSCs were divided into three groups (a control group disposed with DMSO, and two experimental groups disposed with 5 μM Wnt-C59 or 20 μM Wnt-C59), which were all incubated for 24 h. The media of the transwell experiment in both the upper and lower chambers were all disposed with the same concentration as described above. Moreover, the concentrations of DMSO in the media of three groups were guaranteed to be equal. To assess the migration of GSCs, all the procedures were consistent with invasion except that the chamber was not covered with Matrigel and the incubation time was shortened to 8 h. Each assay was performed in triplicate.

### Intracranial and subcutaneous xenograft models and magnetic resonance imaging (MRI)

All animal procedures were approved by the Experimental Animal Ethics Committee of the Cancer Hospital, Chinese Academy of Medical Sciences. For the establishment of intracranial xenograft models, male 4- to 5-week-old BALB/c-nu mice (Beijing HFK Bioscience Ltd) were stabilized with a stereotactic apparatus (KOPF940) with continuous anesthetization with isoflurane (RWD Life Science). Then the shCtrl and shLGR5 GSCs were injected into the caudate nucleus of brain as previously described with 5 × 10^5^ cells in 3 μL of DMEM/mice for U251 or 1 × 10^6^ cells in 5 μL DMEM/mice for 8591. After injection, 5 of 10 shCtrl mice were treated with 200 μL Wnt-C59 (15 mg/kg/day) by oral administration. Then, the intracranial tumor of T2-weight (T2W) images of the shCtrl (*n* = 5), shLGR5 (*n* = 5) and Wnt-C59 (*n* = 5) groups were examined by a 7.0 T MRI scanner (Bruker BioSpin, Billerica, MA, USA) with a relaxation enhancement (RARE) pulse sequence (TR = 4030 ms, TE = 50 ms, matrix = 320 × 384, slice thickness = 0.3 mm). The maximal anteroposterior diameter (L), transverse diameter (W) and height (H) were measured with a RadiAnt DICOM Viewer. Tumor volume was calculated with the following formula: V = π/6 × L × W × H (mm^3^).

For the establishment of subcutaneous xenograft models, the mice and the anesthesia method were the same as with the intracranial xenograft models. The sorting glioma cells suspended in 100 μL of DMEM were injected directly into the dorsal areas (left for LGR5^−^ and right for LGR5^+^) of mice with 5 × 10^5^ cells/mice for U251 or 1 × 10^6^ cells/mice for 8591. We measured the tumor size every 3 days and photographed at the endpoint of the experiment.

### Western blot

Proteins were extracted using a Total Protein Extraction Kit (Invent Biotechnologies, SD-001) according to the manufacturer’s instruction. Western blot analyses were performed as previously reported [34]. The following primary antibodies were used to detect protein expression: LGR5 (#LS-C98619; Biosciences), CD133 (#ab19898; Abcam), CD24 (#ab179821; Abcam), EpCAM (#ab71916; Abcam), CD44 (#3570; Cell Signaling), N-cadherin (#13116; Cell Signaling), E-cadherin (#14472; Cell Signaling), SOX2 (#3579; Cell Signaling), Nanog (#3580; Cell Signaling), OCT4 (#2750; Cell Signaling), Vimentin (#3932; Cell Signaling), β-catenin (#8480; Cell Signaling), phospho-β-catenin^Ser33/37/Thr41^ (#9561; Cell Signaling), Non-phospho (Active) β-catenin ^Ser33/37/Thr41^ (Active-β-catenin) (#8814; Cell Signaling) and β-actin (#4970; Cell Signaling). The dilution was performed according to the antibody specification.

### Immunohistochemistry (IHC) and immunofluorescence (IF)

IHC staining of human glioma specimens and subcutaneous or intracranial tumors were performed as previously described [34]. All antibodies used were as follows: LGR5 (#TA502948; OriGene), Ki67 (#ab16667; Abcam), CD44 (#15675–1-AP, Proteintech), SOX2 (#ab92494; Abcam), Non-phospho (Active) β-catenin ^Ser33/37/Thr41^ (#8814; Cell Signaling) and N-cadherin (#bs-1172R; Bioss). For quantitative analysis of IHC, each slice was randomly photographed from five different fields. The expression levels of LGR5, Ki67, Active-β-catenin, and N-cadherin were analyzed using Image-Pro Plus 5.0. For evaluation of IHC staining of LGR5, the IHC intensity and the expression level of LGR5 were independently reviewed by two observers in a blinded manner. The IHC score was calculated by multiplying the IHC staining intensity and the percentage of positive tumor cells. The staining intensity was defined as follows: 0 = no staining; 1 = weak staining; 2 = medium staining; 3 = strong staining. The percentage of positive tumor cells as graded as 1 for 0–25%, 2 for 26–50%, 3 for 51–75% and 4 for > 75%. The final score of LGR5 staining was used to determine the expression level of LGR5, which was categorized into low expression group (LGR5^low^, total score ≦ 4) and high expression group (LGR5^high^, total score > 5).

IF staining were performed as previously described [35]. The primary antibodies used were as follows: LGR5 (#LS-C98619; Biosciences), LGR5 (#TA502948; OriGene), GFAP (#ab190288; Abcam), SOX2 (#3579; Cell Signaling) and N-cadherin (#13116; Cell Signaling). Alexa Fluor 488 (#4412; Cell Signaling) and Alexa Fluor 647 (#4410; Cell Signaling) were used as the secondary antibody at a dilution of 1:500. IF images were captured with a confocal microscope (BD Biosciences).

### Statistical analysis

Statistical analysis was performed using GraphPad Prism 5.0 (GraphPad Software Inc.) and SPSS version 19.0 (SPSS Inc.). Two-tailed Student *t* test was used to analyze the differences in the results between groups. Comparisons among three or more groups were assessed using a one-way analysis of variance (ANOVA). Comparison between two or more groups in different time points were assessed by two-way ANOVA. Correlations between LGR5 and Ki67, N-cadherin, SOX2 and CD44 expressions were analyzed by Spearman correlation method. All values are expressed as means ± SD. The correlation between LGR5 expression and clinicopathological variables was analyzed by a χ2 test or Fisher’s exact test. OS and PFS curves were plotted by the Kaplan-Meier method and compared using the results of a Log-rank test. The Cox proportional hazards model was used to estimate the independent prognostic factors for OS and PFS in the multivariate analysis. *P* values less than 0.05 were considered statistically significant.

## Results

### Percentage of LGR5^+^ cells is higher in enriched cells than that in parent cells

**Fig. 1:**
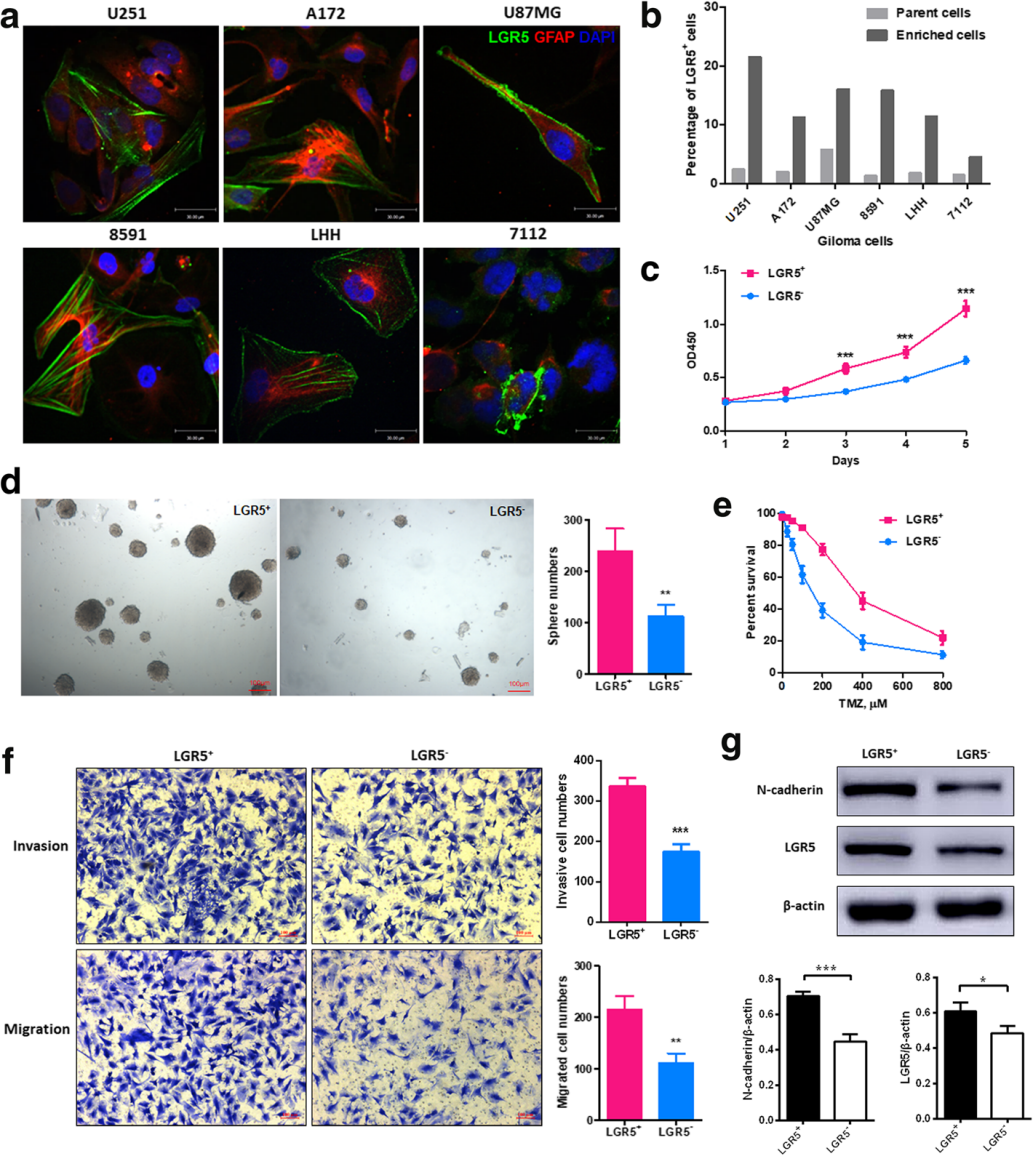
LGR5 expression in different glioma cells, and stemness properties of LGR5^+^ U251 cells in vitro. **a** The expression and localization of LGR5 and GFAP in the glioma cell lines (U251, A172 and U87MG) and the human primary glioma cells (8591, LHH and 7112). Scale bar = 30 μm. **b** The enrichment levels of LGR5 expression by FCM in parent cells and enrichment cells. **c** Cell proliferation assays of LGR5^+^ U251 cells and LGR5^−^ U251 cells (*P* < 0.001, *n* = 3, two-way ANOVA). **d** Coloning sphere images and sphere numbers in the clone formation assay (*P* < 0.01, *n* = 3, Student *t* test). Scale bar = 100 μm. **e** Drug resistance curve of TMZ in LGR5^+^ and LGR5^−^ U251 cells. **f** Images and numbers of invasive cells in invasion assays (top, *P* < 0.001, *n* = 3, Student *t* test) and images and numbers of migrated cells in migration assays (bottom, *P* < 0.001, *n* = 3, Student *t* test). **g** Western blot analysis for LGR5 and N-cadherin in LGR5^+^ and LGR5^−^ U251 cells. The expression levels of LGR5 and N-cadherin were quantified by Image lab software by densitometric analysis and were normalized to the control groups. Human β-actin was used as the internal control. (*P* < 0.05, *n* = 3; *P* < 0.001, *n* = 3, respectively, Student *t* test). All data are represented as mean ± SD from triplicate wells. **, *P* < 0.01; ***, *P* < 0.001, as compared to control

To determine the expression and localization of LGR5 in glioma cells, LGR5 staining was performed in 3 types of glioma cell lines (U251, U87 and A172) and 3 types of human primary glioma cells (8591, LHH and 7112), demonstrating that LGR5 was expressed in the cell membrane and cytoplasm (Fig. 1a). All of the abovementioned glioma cells were proved to be derived from astrocytes by glial fibrillary acidic protein (GFAP) co-dyeing (Fig. 1a). We obtained enriched cells from parent cells through serum-free enrichment, which is a method for quickly screening GSCs. The expression of LGR5 was detected in glioma parent cells and in enriched cells by FCM (Additional file 5: Figure S1a). The positive proportions of LGR5 were 2.46%, 2.01%, 5.76%, 1.34%, 1.79% and 1.45% in U251, U87, A172, 8591, LHH and 7112 parent cells, respectively, and 21.50%, 11.23%, 16.04%, 15.42%, 11.41% and 4.53% in U251, U87, A172, 8591, LHH and 7112 enriched cells, respectively. The positive rates of LGR5 in enriched cells were 8.7, 5.6, 2.8, 11.5, 6.4 and 3.1 times higher than those in parent cells for U251, U87, A172, 8591, LHH and 7112, respectively. Thus, we chose U251 and 8591, whose enrichment levels were the highest (Fig. 1b), to establish cell models. Then, LGR5^+^ and LGR5^−^ cells were obtained by FACS to perform follow-up experiments (Additional file 5: Figure S1b).

### LGR5^+^ cells possess higher proliferation and clone formation abilities

Compared with LGR5^−^ cells, LGR5^+^ U251 cells possessed higher proliferation activity (*P* < 0.001, *n* = 3, Fig. 1c) and presented a higher ability to form colonies (*P* < 0.01, *n* = 3, Fig. 1d). Moreover, the clone formation assay showed that LGR5^+^ U251 cells formed larger spheres, whereas the LGR5^−^ U251 cells formed smaller spheres at day 14 (Fig. 1d). Similar results were obtained for 8591 (Additional file 6: Figure S2a, b). These results indicated that LGR5^+^ cells exhibited stronger tumor initiation and cloning abilities compared to LGR5^−^ cells in vitro.

### LGR5^+^ cells are more resistant to temozolomide (TMZ)

GSCs usually show strong resistance to chemotherapy. TMZ is the foremost medicine for clinical adjuvant treatment of glioma; therefore, we tested the resistance of LGR5^+^ and LGR5^−^ cells to TMZ. The IC_50_ values were 142.5 μM for LGR5^−^ U251 cells and 376.0 μM for LGR5^+^ U251 cells (*n* = 3, Fig. 1e) and were 256.8 μM for LGR5^−^ 8591 cells and 471.3 μM for LGR5^+^ 8591 cells (Additional file 6: Figure S2c). LGR5^+^ glioma cells expressed stronger resistance to TMZ than LGR5^−^ glioma cells.

### LGR5^+^ cells show higher ability of invasion and migration

We performed invasion and migration assays of LGR5^+^ and LGR5^−^ U251 cells and found that LGR5^+^ U251 cells were significantly more invasive and migratory than LGR5^−^ cells (*P* < 0.001, *n* = 3; *P* < 0.001, *n* = 3, respectively, Fig. 1f). Similar results were observed in 8591 primary glioma cells (Additional file 6: Figure S2d). Then, we examined the expression of LGR5 and EMT activated marker N-cadherin in LGR5^+^ and LGR5^−^ cells by Western blot. The results clearly showed higher expression levels of LGR5 and N-cadherin in LGR5^+^ cells (Fig. 1g), suggesting that LGR5 may be related to invasiveness and EMT in glioma.

### Xenograft tumors derived from LGR5^+^ cells are more malignant and invasive, exhibiting strong stemness phenotype

**Fig. 2:**
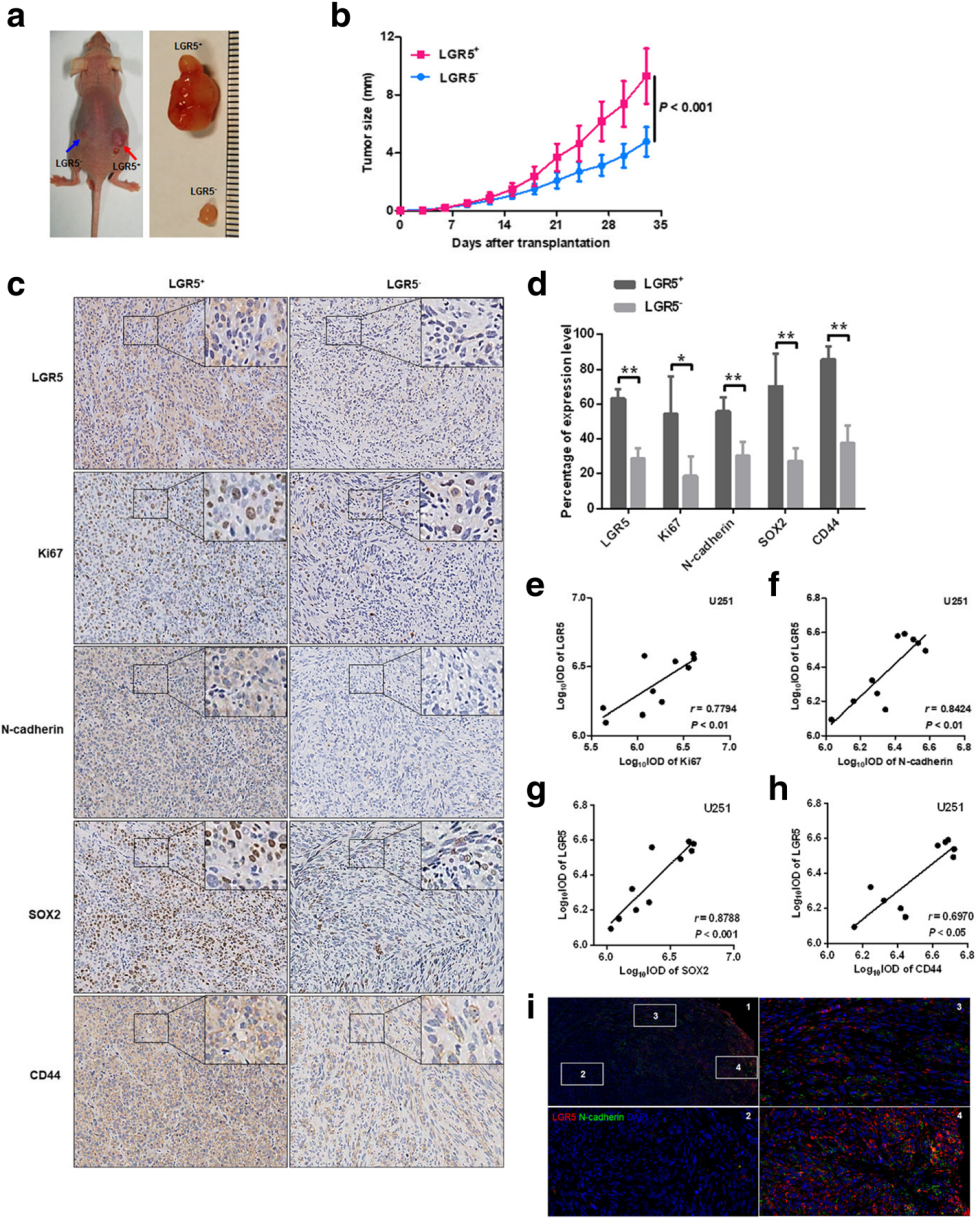
Stemness properties of LGR5^+^ U251 cells in vivo. **a** Xenografts produced by LGR5^+^ and LGR5^−^ U251 cells. **b** The subcutaneous xenografts growth curve generated by LGR5^+^ and LGR5^−^ U251 cells in BALB/c-nu mice (two-way ANOVA, *P* < 0.001). Data are shown as the mean ± SD (LGR5^+^ U251: *n* = 5, LGR5^−^ U251: *n* = 5). **c** IHC staining of LGR5, Ki67, N-cadherin, CD44 and SOX2 expression in LGR5^+^ xenografts and LGR5^−^ xenografts (magnification × 200). **d** The percentage of positive expression of LGR5, Ki67, N-cadherin, CD44 and SOX2 in LGR5^+^ xenografts and LGR5^−^ xenografts (*n* = 5, Student *t* test). Error bars represent the mean ± SD. **e** The correlation between the levels of LGR5 and Ki67 by Spearman correlation analysis (*P* < 0.01, *r* = 0.7794, *n* = 10). **f** The correlation between the levels of LGR5 and N-cadherin by Spearman correlation analysis (*P* < 0.01, *r* = 0.8424, *n* = 10). **g** The correlation between the levels of LGR5 and SOX2 by Spearman correlation analysis (*P* < 0.001, *r* = 0.8788, *n* = 10). **h** The correlation between the levels of LGR5 and CD44 by Spearman correlation analysis (*P* < 0.05, *r* = 0.6970, *n* = 10). **i** Double-staining of LGR5 and N-cadherin in U251 subcutaneous xenografts. The xenografts edge (3 and 4) showed many positive cells expressed both LGR5 and N-cadherin, while there was few positive cells in inside xenografts (section “Methods”). Scale bar = 100 μm. *, *P* < 0.05; **, *P* < 0.01; ***, *P* < 0.001

Tumorigenic ability in vivo is known to be the gold standard for identifying GSCs. We transplanted 5 × 10^5^ LGR5^+^ and 5 × 10^5^ LGR5^−^ U251 cells into the backs of BALB/c-nu mice. The xenografts produced by the LGR5^+^ U251 cells (LGR5^+^ xenografts) were larger than those xenografts produced by the LGR5^−^ U251 cells (LGR5^−^ xenografts) (*P* < 0.001, *n* = 5, Fig. 2a, b). In addition, IHC was performed to validate the role of LGR5 in the malignancy and invasiveness and stemness of glioma xenografts. The expression of LGR5 was remarkably higher in LGR5^+^ xenografts than that in LGR5^−^ xenografts (*P* < 0.01, *n* = 5, Fig. 2c, d). The expression level of Ki67, a sign of proliferation for tumor malignancy, was higher in LGR5^+^ xenografts than that in LGR5^−^ xenografts (*P* < 0.05, *n* = 5, Fig. 2c, d). Moreover, the LGR5^+^ xenografts exhibited significantly higher expression of N-cadherin compared with the LGR5^−^ xenografts (*P* < 0.01, *n* = 5, Fig. 2c, d), consistent with the previous Western blot analysis in LGR5^+^ U251 cells. Notably, the CSCs markers levels of both SOX2 and CD44 were also found to be overexpressed in LGR5^+^ xenografts (*P* < 0.01, *n* = 5; *P* < 0.01, *n* = 5, respectively, Fig. 2c, d). To verify whether LGR5 level correlated with these tumor markers in glioma, Spearman correlation analyses were performed. Significant positive correlations were observed between the LGR5 level and both Ki67 (*P* < 0.01, *r* = 0.7794, *n* = 10, Fig. 2e) and N-cadherin level (*P* < 0.01, *r* = 0.8424, *n* = 10, Fig. 2f), as well as SOX2 level (*P* < 0.001, *r* = 0.8788, *n* = 10, Fig. 2g) and CD44 level (*P* < 0.05, *r* = 0.6970, *n* = 10, Fig. 2h). Additionally, the co-expression of LGR5 and N-cadherin were observed mostly in the peripheral xenograft edge, while positive expression is rare in inside xenografts (Fig. 2i). Similar results were obtained for 8591 cells (Additional file 7: Figure S3). These results suggest that LGR5^+^ cells possess stronger tumorigenicity in vivo than LGR5^−^ cells, and LGR5 may thus be implicated in the malignancy, invasiveness and stemness of glioma.

### Relationship between LGR5 and reported CSC markers and stem cell genes

**Fig. 3:**
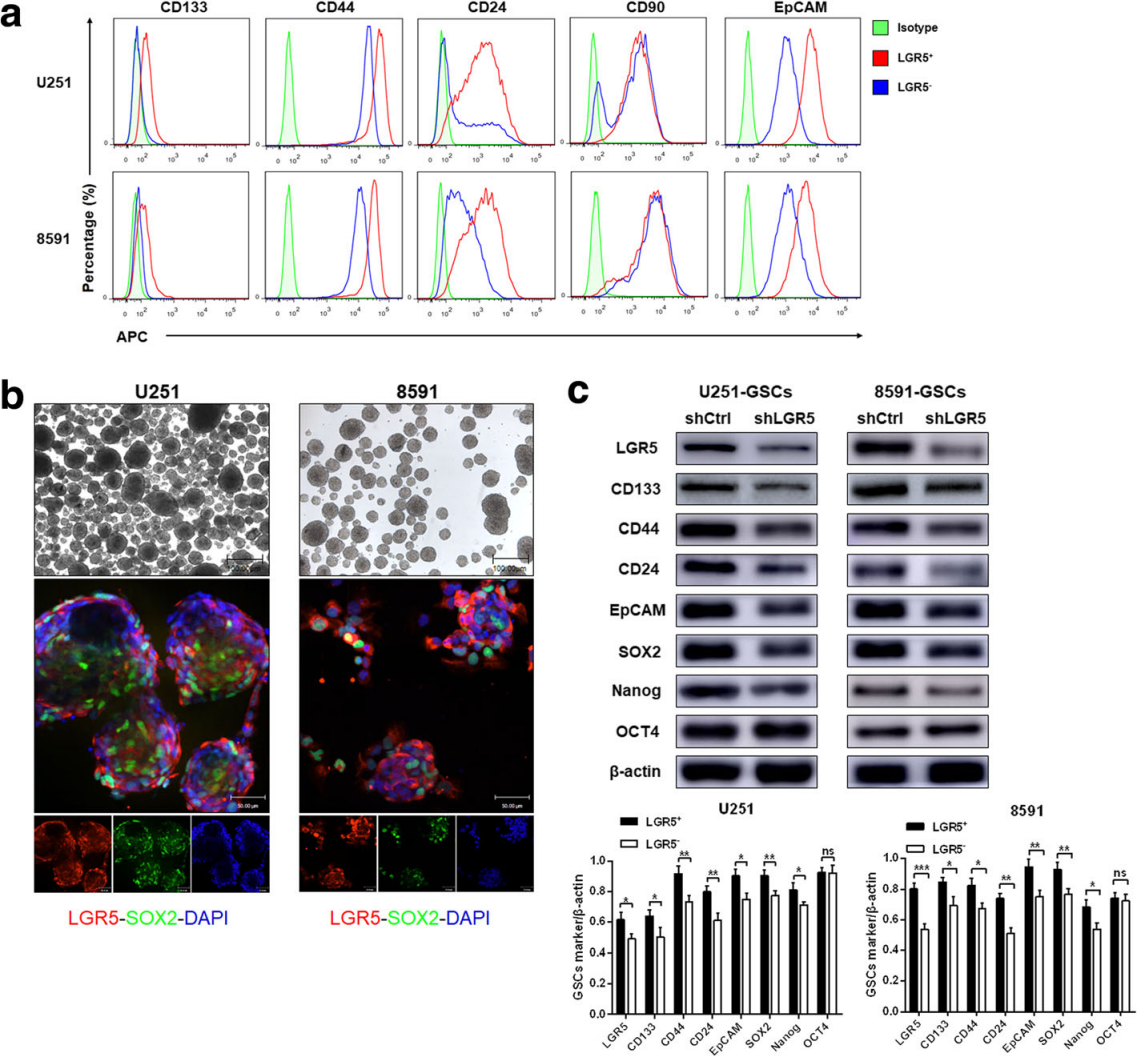
Relationship between LGR5 and reported CSCs markers and stem cell genes. **a** Analyses of CD133, CD44, CD90, CD24, and EpCAM expression in LGR5^+^ and LGR5^−^ U251 glioma cells and in LGR5^+^ and LGR5^−^ 8591 human primary glioma cells by FCM. **b** U251-GSCs and 8591-GSCs from U251 and 8591 human primary glioma cells through serum-free enrichment (top, scale bar = 100 μm) and immunofluorescence double staining for LGR5 and SOX2 (bottom, scale bar = 50 μm). **c** The expression levels of LGR5, CSCs markers (CD133, CD44, CD24 and EpCAM) and stem cell genes (SOX2, OCT4 and Nanog) in shLGR5 and shCtrl GSCs determined by Western blot (top). The protein intensity of these markers were determined by densitometric analysis and normalized to the relevant β-actin value. Error bars represent the mean ± SD of three independent experiments (bottom). *, *P* < 0.05; **, *P* < 0.01; ***, *P* < 0.001

The expression levels of CD133, CD44, CD24, CD90 and EpCAM were examined in LGR5^+^ and LGR5^−^ U251 and 8591 glioma cells by FCM. We found that LGR5^+^ glioma cells possessed considerably enhanced expression of CD133, CD44, CD90, CD24, and EpCAM (Fig. 3a). Conversely, LGR5^−^ glioma cells showed low expression of CD133, CD44, CD90, CD24, and EpCAM, while the expression of CD90 remained unchanged in LGR5^+^ and LGR5^−^ glioma cells (Fig. 3a).

To further reveal the relationship between LGR5 and the reported CSC markers in GSCs, we obtained enriched cells (U251-GSCs, 8591-GSCs) from U251 and 8591 glioma cells (Fig. 3b, top). Immunofluorescence double staining showed that LGR5 and SOX2 were co-expressed in both U251-GSCs and 8591-GSCs (Fig. 3b, bottom). Then, U251-GSCs and 8591-GSCs were infected with a lentiviral shRNA vector targeting LGR5 (shLGR5) and a control shRNA (shCtrl). The infection efficiency was determined by Western blot (Fig. 3c) and FCM (Additional file 8: Figure S4). We determined that the expression levels of CSC markers CD133, CD44, CD24 and EpCAM in shLGR5 GSCs were significantly decreased compared with those of the shCtrl GSCs in both U251 and 8591 GSCs (Fig. 3c). Furthermore, stem cell genes Sox2, OCT4 and Nanog, critical factors favoring stem cell maintenance [10], were also examined. Although the expression of OCT4 remain unchanged, the expression levels of SOX2 and Nanog were obviously decreased in shLGR5 GSCs (Fig. 3c), suggesting that LGR5 might play a prominent role in the maintenance of GSCs.

### LGR5 promotes invasion, migration and EMT in GSCs

**Fig. 4:**
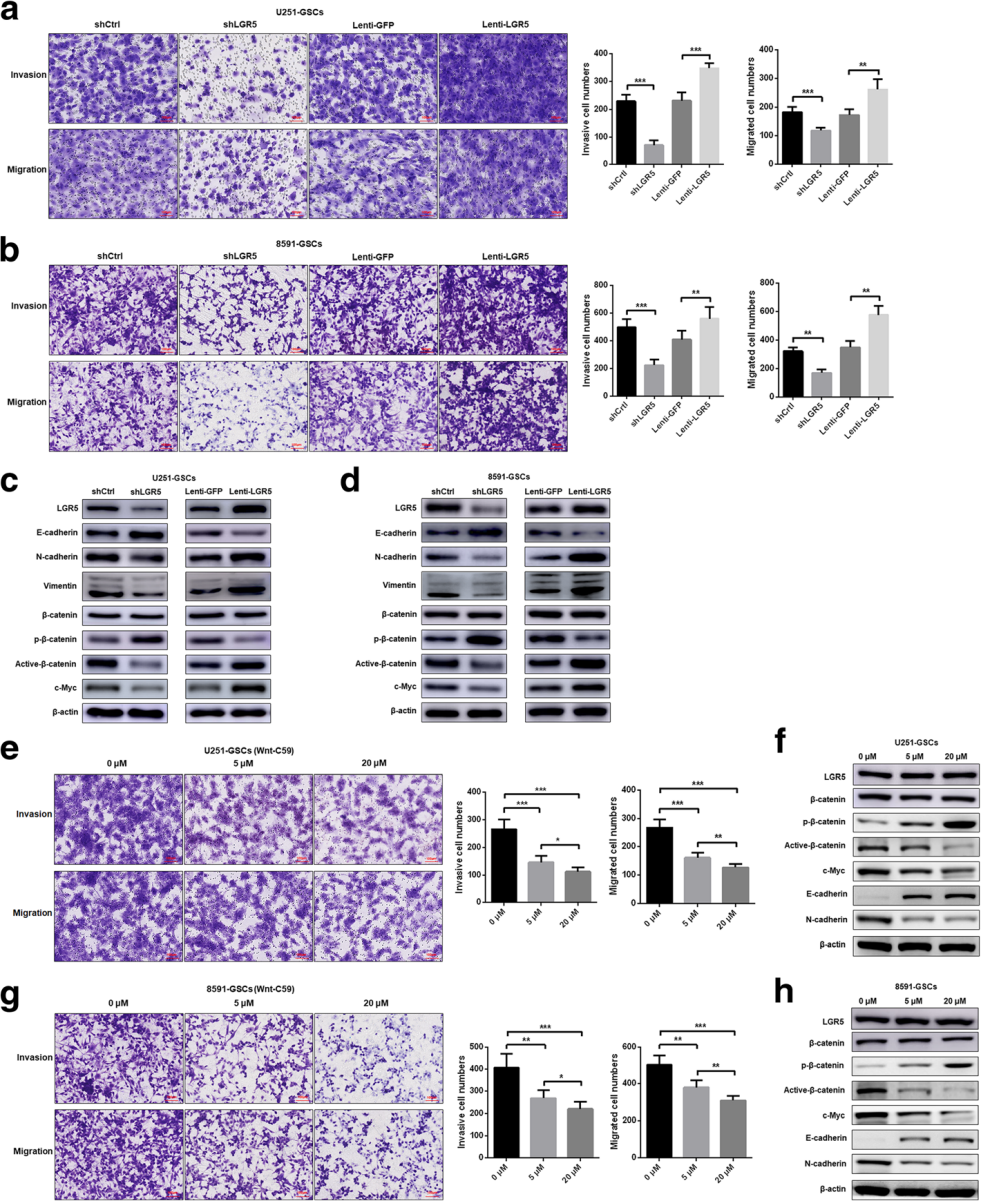
Relationship between LGR5 and EMT and the Wnt/β-catenin pathway, and Wnt-C59 treatment in GSCs. **a** Images and statistical results in invasion assays (*P* < 0.001, *n* = 3; *P* < 0.001, *n* = 3, Student *t* test for shLGR5 and Lenti-LGR5, respectively) and migration assays (*P* < 0.001, *n* = 3; *P* < 0.01, *n* = 3, Student *t* test for shLGR5 and Lenti-LGR5, respectively) for transfected U251 GSCs. **b** Images and statistical results in invasion assays (*P* < 0.001, *n* = 3; *P* < 0.01, *n* = 3, Student *t* test for shLGR5 and Lenti-LGR5, respectively) and migration assays (*P* < 0.01, *n* = 3; *P* < 0.01, *n* = 3, Student *t* test for shLGR5 and Lenti-LGR5, respectively) for transfected 8591 GSCs. **c**, **d** Western blot analyses of transfected U251 and 8591 GSCs for LGR5, EMT related genes and surrogate markers for the activated Wnt/β-catenin pathway. **e** Images and statistical results of invasion assays (*P* < 0.001, *n* = 3; *P* < 0.001, *n* = 3; *P* < 0.05, *n* = 3, one-way analysis of variance (ANOVA) for 0 μΜ and 5 μM, 0 μΜ and 20 μM and 5 μM and 20 μM, respectively) and migration assays (*P* < 0.001, *n* = 3; *P* < 0.001, *n* = 3; *P* < 0.01, *n* = 3, one-way analysis of variance (ANOVA) for 0 μΜ and 5 μM, 0 μΜ and 20 μM and 5 μM and 20 μM, respectively) at 24 h after treated with Wnt-C59 in U251-GSCs. **f** Western blot analyses of U251 GSCs for LGR5, EMT related genes and surrogate markers for the activated Wnt/β-catenin pathway at 48 h after treated with Wnt-C59. **g** Images and statistical results of invasion assays (*P* < 0.01, *n* = 3; *P* < 0.001, *n* = 3; *P* < 0.05, *n* = 3, one-way analysis of variance (ANOVA) for 0 μΜ and 5 μM, 0 μΜ and 20 μM and 5 μM and 20 μM, respectively) and migration assays (*P* < 0.01, *n* = 3; *P* < 0.001, *n* = 3; *P* < 0.01, *n* = 3, one-way analysis of variance (ANOVA) for 0 μΜ and 5 μM, 0 μΜ and 20 μM and 5 μM and 20 μM, respectively) at 24 h after treated with Wnt-C59 in 8591-GSCs. **h** Western blot analyses of 8591 GSCs for LGR5, EMT related genes and surrogate markers for the activated Wnt/β-catenin pathway at 48 h after treated with Wnt-C59. Scale bar = 100 μm. All bars are represented as mean ± SD from triplicate wells. ***, *P* < 0.001; **, *P* < 0.01; *, *P* < 0.05

Previous experiments have shown that LGR5^+^ cells possess stemness characteristics, particularly aggressive characteristics related to EMT. With the primary focus on strong invasion ability and N-cadherin expression of LGR5^+^ cells, we explored the association between LGR5 and EMT in GSCs. The LGR5-overexpressed GSCs (Lenti-LGR5) and their control group (Lenti-GFP) were obtained by lentivirus transfection with U251-GSCs and 8591-GSCs. The transfection efficiency was determined by Western blot (Fig. 4c, d) and FACS (Additional file 8: Figure S4). Thus, invasion and migration assays were performed in shLGR5 and Lenti-LGR5 groups separately as well as in the controls (shCtrl and Lenti-GFP). We found that the invasion and migration capacities of shLGR5 GSCs were weakened as a result of LGR5 knockdown, whereas the invasion and migration capacities of Lenti-LGR5 GSCs were enhanced as a result of LGR5 overexpression (Fig. 4a, b).

To determine whether LGR5 is involved in EMT, we determined the expression levels of E-cadherin, N-cadherin and Vimentin in the abovementioned transfected GSCs. The expression levels of the EMT markers were lower in the shLGR5 GSCs compared with those in the shCtrl GSCs and higher in the Lenti-LGR5 GSCs compared with those in the Lenti-GFP GSCs (Fig. 4c, d). These results indicate that LGR5 can promote the EMT of GSCs.

### LGR5 promotes EMT by activating Wnt/β-catenin signaling in GSCs

The active form of β-catenin (active β-catenin) which is free from degraded by phosphorylation and proteolytic enzymes represents the activated Wnt/β-catenin pathway [36, 37]. We verified the relationship between LGR5 and β-catenin phosphorylation. As shown in Fig. 4c and d, the expression level of phospho-β-catenin ^Ser33/37/Thr41^ (p-β-catenin) was up-regulated and that of Non-phospho (Active) β-catenin ^Ser33/37/Thr41^ (Active-β-catenin) was down-regulated in the shLGR5 GSCs, while the expression level of total β-catenin remained unaltered. Conversely, Lenti-LGR5 GSCs showed decreased expression of p-β-catenin, increased expression of Active-β-catenin and unchanged expression of total β-catenin compared with the Lenti-GFP GSCs (Fig. 4c, d). As expected, the expression of c-Myc, a downstream target gene of the Wnt/β-catenin pathway [38], was proved to be modulated by LGR5 (Fig. 4c, d). These results confirmed that LGR5 can activate the Wnt/β-catenin pathway by inhibiting β-catenin phosphorylation in GSCs.

Based on the previous results indicating that LGR5 can promote EMT, we assumed that LGR5 promotes EMT by activating the Wnt/β-catenin pathway. To test this assumption, we treated U251 and 8591 GSCs with the Wnt/β-catenin inhibitor Wnt-C59 and found that the expression levels of both the Wnt/β-catenin pathway and EMT markers were inhibited in U251 and 8591 GSCs except for LGR5 expression level (Fig. 4f, h). Likewise, the invasion and migration capacities of U251 and 8591 GSCs were both decreased in a dose-dependent manner (Fig. 4e, g). Overall, we demonstrated that LGR5 can promote EMT and enhance the invasion and migration capacity of GSCs by activating the Wnt/β-catenin pathway (Fig. 6h).

### Knockdown of LGR5 and Wnt-C59 treatment inhibit growth of intracranial orthotopic xenografts and prolong overall survival (OS) of xenograft mice

**Fig. 5:**
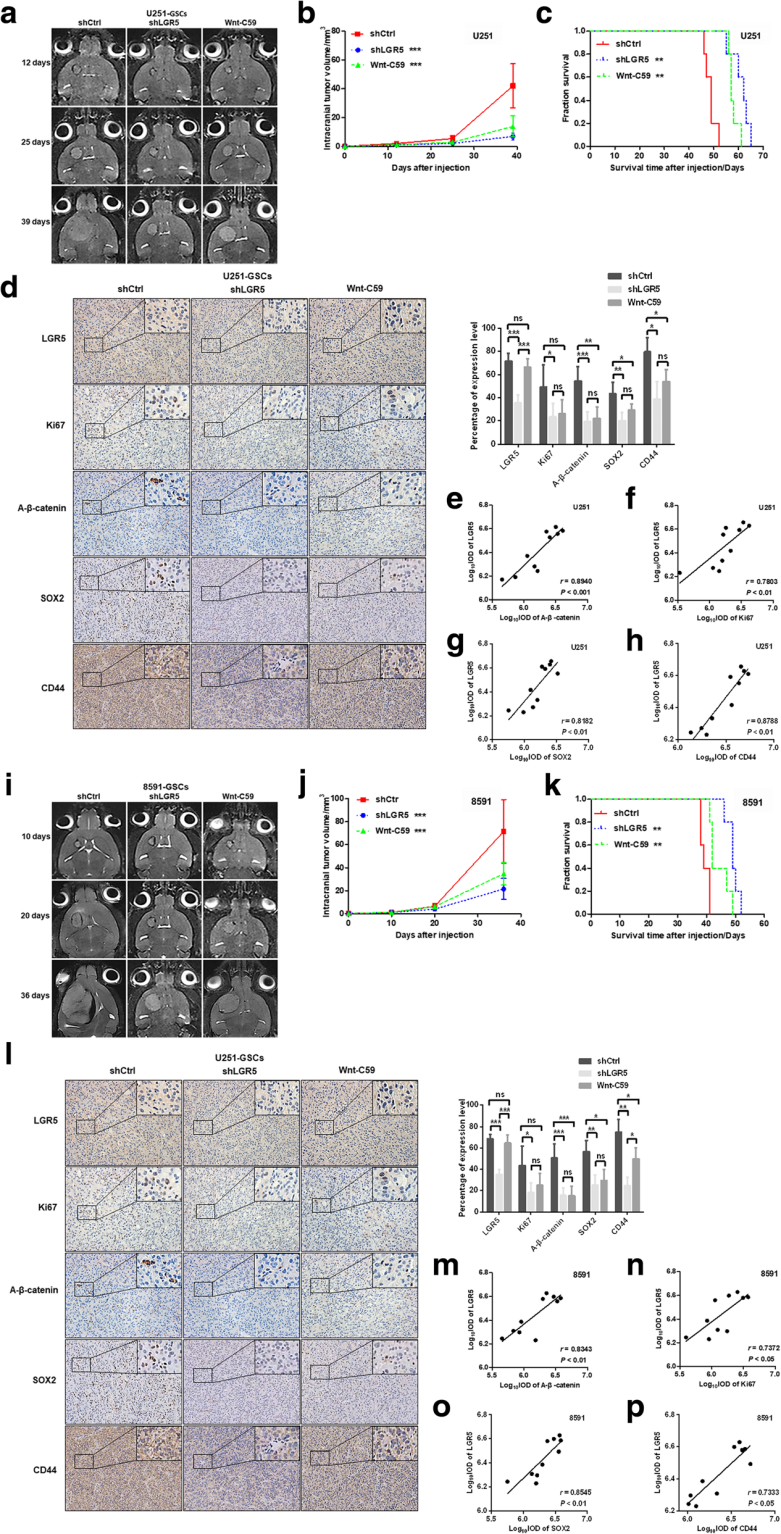
Effect of LGR5 on intracranial tumor growth and overall survival time of xenograft mice. **a** T2W images of intracranial tumor in coronal views scanned using 7.0 T MRI on days 12, 25 and 39 after U251-GSCs injection. **b** The intracranial tumor growth curve of the shLGR5 (*n* = 5), Wnt-C59 (*n* = 5) and shCtrl (*n* = 5) U251-GSCs. Error bars represent the mean ± SD (*n* = 5, two-way ANOVA). **c** Overall survival time (OS) of xenograft mice in the shLGR5 (*n* = 5), Wnt-C59 (*n* = 5) and shCtrl (*n* = 5) U251-GSCs analyzed by Log-rank test. **d** IHC staining and quantitative analyses of LGR5, Ki67, Active-β-catenin, SOX2 and CD44 (magnification × 200). **e** Spearman correlation analysis of LGR5 and Active-β-catenin between shLGR5 and shCtrl U251-GSCs (*P* < 0.001, *r* = 0.8940, *n* = 10). **f** Spearman correlation analysis of LGR5 and Ki67 between shLGR5 and shCtrl U251-GSCs (*P* < 0.01, *r* = 0.7803, *n* = 10). **g** Spearman correlation analysis of LGR5 and SOX2 between shLGR5 and shCtrl U251-GSCs (*P* < 0.01, *r* = 0.8182, *n* = 10). **h** Spearman correlation analysis of LGR5 and CD44 between shLGR5 and shCtrl U251-GSCs (*P* < 0.01, *r* = 0.8788, *n* = 10). **i** T2W images of intracranial tumor in coronal views scanned using 7.0 T MRI on days 10, 20 and 36 after 8591-GSCs injection. **j** The intracranial tumor growth curve of the shLGR5 (*n* = 5), Wnt-C59 (*n* = 5) and shCtrl (*n* = 5) 8591-GSCs. Error bars represent the mean ± SD (*n* = 5, two-way ANOVA). **k** Overall survival time (OS) of xenograft mice in the shLGR5 (*n* = 5), Wnt-C59 (*n* = 5) and shCtrl (*n* = 5) 8591-GSCs analyzed by Log-rank test. **l** IHC staining and quantitative analyses of LGR5, Ki67, Active-β-catenin, SOX2 and CD44 (magnification × 200). **m** Spearman correlation analysis of LGR5 and Active-β-catenin between shLGR5 and 8591-GSCs (*P* < 0.01, *r* = 0.8343, *n* = 10). **n** Spearman correlation analysis of LGR5 and Ki67 between shLGR5 and shCtrl 8591-GSCs (*P* < 0.05, *r* = 0.7372, *n* = 10). **o** Spearman correlation analysis of LGR5 and SOX2 between shLGR5 and shCtrl 8591-GSCs (*P* < 0.01, *r* = 0.8545, *n* = 10). **p** Spearman correlation analysis of LGR5 and CD44 between shLGR5 and shCtrl 8591-GSCs (*P* < 0.05, *r* = 0.7333, *n* = 10). ***, *P* < 0.001; **, *P* < 0.01; *, *P* < 0.05; ns, not significant

Gliomas are different from other tumors due to the presence of the blood-brain barrier. Therefore, it is essential to treat glioma in the intracranial orthotopic xenograft model. We thus conducted a therapeutic experiment using the intracranial xenograft model in three groups, shCtrl, shLGR5 and shCtrl by Wnt-C59 treatment (Wnt-C59), to explore the role of LGR5 in vivo. The intracranial tumors induced by U251-GSCs were assessed on days 12, 25 and 39 (Fig. 5a). The corresponding tumor growth curve showed that tumors in the shLGR5 (*n* = 5) and Wnt-C59 (*n* = 5) groups were significantly inhibited, in contrast to those in the shCtrl group (*n* = 5; Fig. 5b, *P* < 0.001, *P* < 0.001, respectively). The median survival times (MS) of mice in the shLGR5 and Wnt-C59 groups were 62 ± 4.45 days and 57 ± 1.83 days, respectively, while in the shCtrl group, the MS was 49 ± 2.15 days. A log-rank test of the three groups revealed that the OS of the shLGR5 and Wnt-C59 mice was significantly prolonged compared with that of the shCtrl mice (*n* = 5; Fig. 5c, *P* < 0.01, *P* < 0.01, respectively). In addition, quantitative analysis of IHC was used to determine the expression of LGR5, Ki67, Active-β-catenin, SOX2 and CD44. The expression level of LGR5 was statistically significant between the shCtrl and shLGR5 groups (*P* < 0.001, Fig. 5d) and between the shCtrl and Wnt-C59 groups (*P* < 0.001, Fig. 5d). Notably, the expression levels of Ki67, Active-β-catenin, SOX2 and CD44 in both the shLGR5 and Wnt-C59 groups were lower than those in the shCtrl group, whereas the expression levels of above markers showed no differences between the shLGR5 and Wnt-C59 groups (Fig. 5d). Furthermore, Spearman correlation tests verified that the expression of LGR5 was positively correlated with Active-β-catenin (*P* < 0.001, *r* = 0.8940, *n* = 10, Fig. 5e) and Ki67 (*P* < 0.01, *r* = 0.7803, *n* = 10, Fig. 5f), as well as SOX2 (*P* < 0.01, *r* = 0.8182, *n* = 10, Fig. 5g) and CD44 (*P* < 0.01, *r* = 0.8788, *n* = 10, Fig. 5h), between the shLGR5 and shCtrl groups. Similar results were observed in 8591 GSCs (Fig. 5i-p). These results demonstrated that the inhibition of LGR5 could inhibit glioma growth and prolong the survival of the xenograft mice in vivo effectively, indicating that LGR5 might serve as a prospective therapeutic target for glioma.

### Expression levels of LGR5 correlate with WHO grades, Ki67 and N-cadherin

**Fig. 6:**
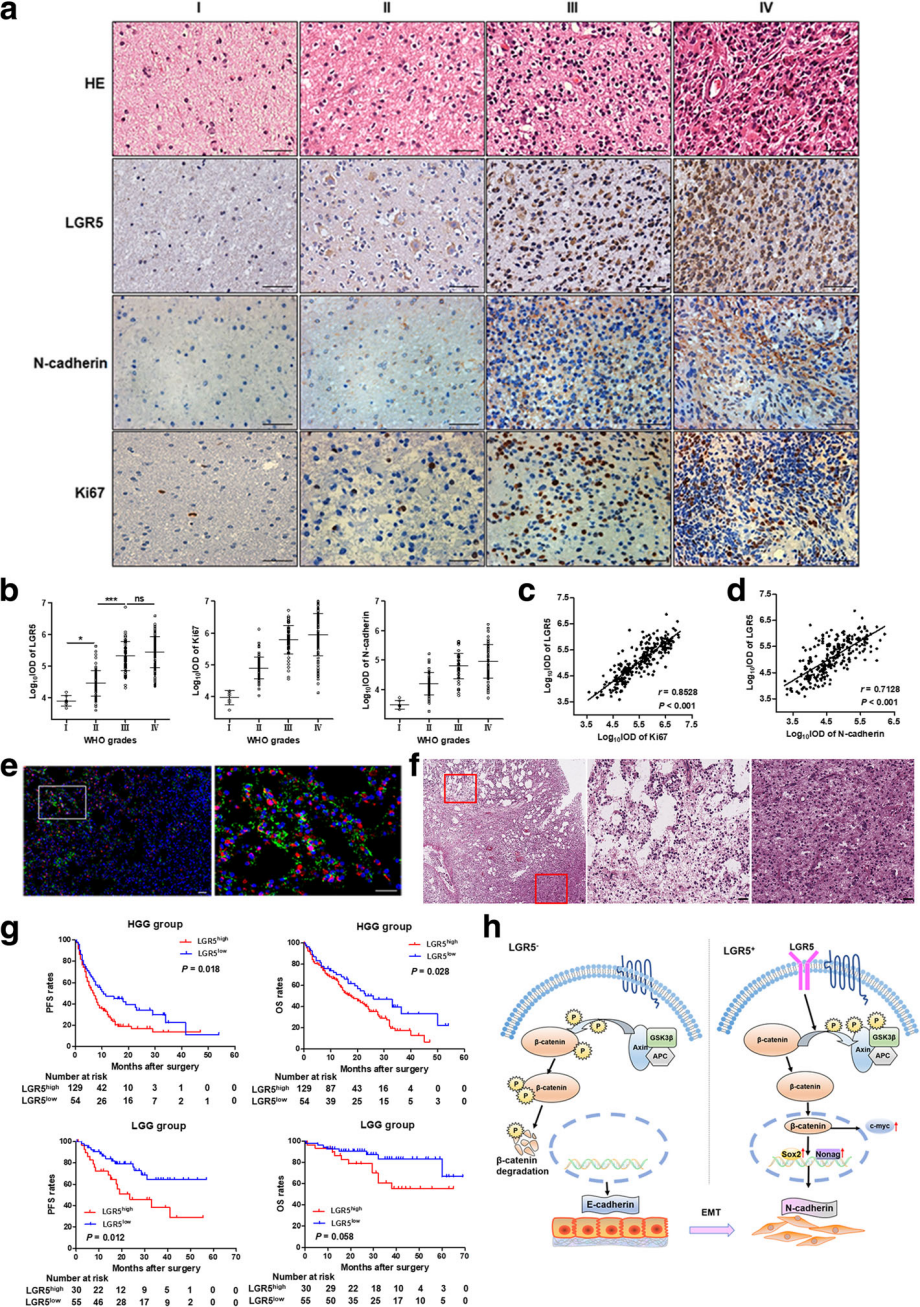
Immunohistochemistry analyses of human glioma specimens. **a** HE and LGR5, Ki67 and N-cadherin staining of human glioma specimens from four patients in four WHO grades. Scale bar = 100 μm. **b** Quantitative analyses of LGR5, Ki67 and N-cadherin in four WHO grades (WHO I and II, *n* = 85; WHO III and IV, *n* = 183, Student *t* test). Data are showed as mean ± SD. **c** Spearman correlation analyses of LGR5 and Ki67 (*P* < 0.001, *r* = 0.8528, *n* = 268). **d** Spearman correlation analyses of LGR5 and N-cadherin (*P* < 0.001, *r* = 0.7128, *n* = 268). **e** LGR5 and N-cadherin staining in glioma tissues. Many glioma cells with high expression of LGR5 and N-cadherin were mostly observed in the invasive edge of tumor (left of 1st picture) compared with the interior of tumor (right of 1st picture). Scale bar = 100 μm. **f** The HE staining of the glioma tissues. The structure of tumor edge (left top), consistent with the invasive edge of Fig. 6e, were more incompact compared with the inside tumor (right bottom). **g** PFS and OS months in low-grade glioma (LGG) group and high-grade glioma (HGG) group according to LGR5 expression in the glioma specimens. ***, *P* < 0.001; *, *P* < 0.05; ns, not significant. **h** A schema diagram displaying the role of LGR5 in regulating Wnt, and EMT in glioma stem cells. Based on the findings of this study, LGR5 could activate Wnt pathway by promoting β-catenin dephosphorylation and result in EMT eventually

The expression levels of LGR5, Ki67 and N-cadherin were determined in 268 human glioma specimens by IHC (Fig. 6a). Significant differences in LGR5 levels were observed between high-grade (WHO III and IV) and low-grade (WHO I and II) glioma as well as between WHO I and WHO II glioma (*P* < 0.001, *P* < 0.05, respectively, Fig. 6b). In addition, both Ki67 and N-cadherin levels increased with grade (Fig. 6b). Spearman correlation analyses were performed to determine whether LGR5 levels correlated with Ki67 and N-cadherin. Positive correlations were observed between the levels of LGR5 and Ki67 (*P* < 0.001, *r* = 0.8528, *n* = 268, Fig. 6c) and between the levels of LGR5 and N-cadherin (*P* < 0.001, *r* = 0.7128, *n* = 268, Fig. 6d). In addition, many glioma cells with both LGR5 and N-cadherin expression were observed in the invasive edge of human glioma tissues, while the internal tumor rarely expressed LGR5 and N-cadherin (Fig. 6e). The HE image showed the tissue structure of tumor edge, consistent with the invasive edge in Fig. 6e, were more incompact compared with the inside tumor (Fig. 6f).

### Correlations of LGR5 levels with survival of glioma patients

In view of the differences in LGR5 expression as well as the multifarious differences in high- and low-grade glioma [2], we divided all glioma patients into a low-grade glioma (LGG, WHO I and II) group and a high-grade glioma (HGG, WHO III and IV) group. The clinicopathologic characteristics of glioma patients and their correlations with LGR5 expression are summarized in Additional file 1: Table S1 and Additional file 2: Table S2. LGR5 expression levels were positively correlated with Ki67 and negatively correlated with isocitrate dehydrogenase 1 (IDH1) in both the HGG group and LGG group (Additional file 1: Table S1 and Additional file 2: Table S2). Notably, although there was no statistical significance, patients with high expression of LGR5 (LGR5^high^) in the HGG group tended to have more severe peritumoral edema (*P* = 0.079) (Additional file 1: Table S1). Representative images of peritumoral edema in the HGG group and the LGG group are shown in Additional file 9: Figure S5.

Log-rank tests were performed to explore whether LGR5 expression level is significant with respect to the prognosis of glioma patients. In the HGG group, LGR5^high^ patients had significantly lower progression-free survival (PFS) (*P* = 0.018) and OS (*P* = 0.028) compared with that of patients with low expression of LGR5 (LGR5^low^). The median PFS and OS were 7.0 and 17.5 months for LGR5^high^ patients and 10.5 and 23.0 months for LGR5^low^ patients, respectively (Additional file 10: Table S3). In the LGG group, LGR5^high^ patients had significantly lower RFS (*P* = 0.012) than LGR5^low^ patients, while the OS was not statistically significant between LGR5^high^ patients and LGR5^low^ patients (*P* = 0.058). The median PFS of LGR5^high^ patients was 23.5 months; the OS of LGR5^high^ patients and the PFS and OS of LGR5^low^ patients in the LGG group were not available (Additional file 11: Table S4).

Furthermore, in the HGG group, univariate analysis revealed that LGR5 expression, peritumoral edema and chemoradiotherapy were correlated with worse PFS (Additional file 10: Table S3). Multivariate Cox regression analysis demonstrated that LGR5 expression and chemoradiotherapy were independent indicators of postoperative recurrence (Table 1). Moreover, LGR5 expression, the Karnofsky Performance Scale (KPS), IDH1 mutation and chemoradiotherapy were associated with worse OS according to the log-rank test (Additional file 10: Table S3). Multivariate analysis showed that LGR5 expression, IDH1 mutation and chemoradiotherapy were independent indicators of OS (Table 1). In the LGG group, IDH1 mutation and chemoradiotherapy significantly decreased both RFS and OS, whereas LGR5 expression was only related with shorter PFS but not OS (Additional file 11: Table S4). In multivariate analysis, LGR5 expression and chemoradiotherapy were key factors resulting in shorter PFS (Table 1).

**Table 1:** Multivariate analysis of factors predicting prognosis

Variable	Hazard Ratio (95% CI)	*P* value
Progression-free survival in the HGG group		
Chemoradiotherapy	0.58 (0.41–0.85)	***0.005***
LGR5	1.64 (1.10–2.43)	***0.012***
Peritumoral edema	1.28 (0.88–1.85)	0.193
Overall survival in the HGG group		
Chemoradiotherapy	0.53 (0.32–0.87)	***0.019***
LGR5	1.65 (1.07–2.53)	***0.024***
IDH1 mutation	0.56 (0.34–0.93)	***0.024***
KPS	0.71 (0.49–1.04)	0.080
Progression-free survival in the LGG group		
Chemoradiotherapy	0.18 (0.05–0.62)	***0.007***
LGR5	2.56 (1.20–5.45)	***0.015***
IDH1 mutation	0.48 (0.23–1.02)	0.056
Overall survival in the LGG group		
IDH1 mutation	0.26 (0.09–0.74)	***0.012***
LGR5	2.08 (0.80–5.42)	0.136
Chemoradiotherapy	0.00 (0.00–2.03)	0.969

Abbreviation: *HGG* high-grade glioma, *LGG* low-grade glioma, *LGR5* leucine-rich repeat-containing G protein-coupled receptor 5, *KPS* karnofsky performance scale, *IDH1* isocitrate dehydrogenase 1, *CI* confidence interval, *NA* not available.

Bold and italic value represents *P* < 0.05 that are considered statistically significant

## Discussion

Most glioma cases tend to show recurrence and poor prognosis, which are believed to be closely related to the existence of GSCs [10–12]. It is necessary to identify GSCs and find GSC-related therapeutic targets to improve glioma outcomes. Our findings showed that LGR5 can serve as a functional marker of GSCs with strong stemness properties. The finding that LGR5 proportion was much higher in enriched cells than that in parent cells suggests that LGR5 conformed to the typical features of a GSC marker. Cell proliferation assays and clone-formation assays revealed stronger malignant potency of LGR5^+^ cells. LGR5^+^ cells were more resistant to TMZ than LGR5^−^ cells. Invasion and migration assays showed that LGR5^+^ cells were more invasive and migratory. Moreover, the tumors produced by LGR5^+^ cells were larger, more invasive and malignant in subcutaneous transplantation experiments than those produced by LGR5^−^ cells. These results strongly indicate that LGR5^+^ glioma cells possess a stronger stem-like phenotype.

Several CSC markers have been reported in glioma, such as CD133, CD24, CD90, CD44 and EpCAM [10, 21, 22]. Our FCM analysis revealed that LGR5^+^ glioma cells displayed significantly higher expression of these CSCs markers, indicating that LGR5^+^ glioma cells have stronger stem cell characteristics than LGR5^−^ cells. Furthermore, the conjecture that the expression of these CSCs markers in GSCs were modulated by LGR5 was confirmed by results indicating reduced expression of CD133, CD24, CD44 and EpCAM in LGR5 knockdown GSCs. In addition, although OCT4 expression did not change, we found that the acknowledged stem cell genes SOX2 and Nanog were obviously regulated by LGR5. These results suggest that LGR5 may serve as a vital and advanced GSC marker.

LGR5 has been reported as a proliferative factor in multiple tumors, with the ability to regulate cell cycle proteins, such as p27, pRb and CyclinD1 [36, 39]. In addition, studies have shown that the expression of LGR5 is important for the cloning and tumorigenic abilities of glioma cells [30, 33]. Similarly, our research showed that LGR5^+^ cells possessed significantly stronger stem cell characteristics in cell proliferation, cloning, tumorigenicity, and even drug resistance. However, the role of LGR5 in glioma invasion remains unclear, particularly for GSCs. Recently, increasing evidences indicated that the contribution of GSCs to tumor recurrence is most likely attributed to its EMT phenotype [8, 19, 40]. Therefore, it is important to find the primary effector molecules and signaling pathways that driven EMT in GSCs.

In this study, for the first time, we found that LGR5 was associated with the invasiveness of GSCs in vitro and in vivo. LGR5 could significantly affect invasion and migration in GSCs. The tumors derived from LGR5^+^ cells possessed higher invasive phenotype. Moreover, the epithelial marker E-cadherin and the mesenchymal markers including N-cadherin and vimentin were regulated by LGR5 in GSCs, suggesting that LGR5 is a key effector molecule that drives the EMT of GSCs. Then, we explored the mechanism of glioma invasion and migration regulated by LGR5. Although LGR5 has been reported to be involved in the Wnt/β-catenin pathway [25], it has not been verified in glioma. We demonstrated that LGR5 can activate the Wnt/β-catenin pathway by inhibiting the phosphorylation of β-catenin in vitro and in vivo. Furthermore, Wnt-C59, a Wnt/β-catenin pathway inhibitor, inhibited both EMT and the Wnt/β-catenin pathway as well as the invasion and migration of glioma cells. Based on the abovementioned loop treatment verification, we can conclude that LGR5 can promote glioma invasion, migration and EMT by activating the Wnt/β-catenin pathway (Fig. 6h).

Notably, we found that both LGR5 knockdown and Wnt-C59 significantly inhibited the growth of tumors and prolonged the survival time of mice in intracranial orthotopic xenograft models. Additionally, the treatment effect of orthotopic xenografts tended to be more distinct in the LGR5 knockdown group, suggesting the possibility of the involvement of LGR5 with other tumorigenesis-related pathways. IHC analysis of orthotopic xenografts revealed that the expression levels of Ki67 and Active-β-catenin were significantly decreased in both the LGR5 knockdown and Wnt-C59 groups. Furthermore, LGR5 expression was significantly correlated with Ki67 and Active-β-catenin. These results illustrate that LGR5 may be a potential target for blocking EMT in GSCs.

Similar to the results of the animal experiment in vivo, LGR5 expression level was proved to be positively correlated with WHO grade, Ki67 and N-cadherin by quantitative analysis of IHC of human glioma tissues. In addition, LGR5^+^ N-cadherin^+^ cells were mostly observed in the invasive front of human glioma tissues, while glioma cells inside tumors rarely expressed LGR5 and N-cadherin, suggesting a correlation between the two markers and their important role in the high invasion of glioma.

Furthermore, IDH1 mutation, a favorable predictor of prognosis [41], was confirmed to be negatively correlated with LGR5 by chi-square tests in both HGG and LGG group. This result indicated that IDH1 mutation in LGR5^high^ glioma occurred less frequently than that in LGR5^low^ glioma, suggesting that glioma patients with high expression of LGR5 may have a poor prognosis. However, the relationship between LGR5 and IDH1 must be further explored. Although LGR5 has been identified as a prognostic factor in many tumors [30, 33, 42, 43], there are few studies on the prognostic value of LGR5 in glioma. The prognostic value of LGR5 in glioma need more evidence to confirm. To verify the value of LGR5 in estimating glioma prognosis, univariate and multivariate analysis were performed. The results revealed that LGR5 expression was an independent indicator of postoperative recurrence in both the HGG group and LGG group; moreover, LGR5 expression was also an independent indicator of OS in the HGG group, consistent with the results indicating that LGR5 knockdown prolonged the survival of nude mice in vivo. However, in the LGG group, LGR5 expression tended to be related to poor OS in univariate analysis (*P* = 0.058). This result may be attributed to the fact that the review period of this study was not sufficiently long. Additionally, LGR5 expression tended to relate to peritumoral edema in HGG group, although the difference was not statistically significant (*P* = 0.079). Overall, our finding that LGR5^high^ expression identifies a subset of glioma patients with a poorer survival profile than LGR5^low^ expression might provide aid for clinicians in determining the prognosis of glioma patients.

## Conclusions

Our study demonstrates that LGR5 can serve as a functional GSC marker, drive GSCs EMT and by activating the Wnt/β-catenin pathway in vitro and in vivo, and predicts glioma recurrence and poor prognosis. Thus, we believe that further studies targeting LGR5 will provide novel therapeutic approaches for treating GSCs.

## Additional files

Additional file 1: **Table S1.** Association of LGR5 expression with clinicopathological features in HGG group. (XLS 10 kb)

Additional file 2: **Table S2.** Association of LGR5 expression with clinicopathological features in LGG group. (XLS 10 kb)

Additional file 3: Short tandem repeat (STR) profiling of U251. (PDF 2190 kb)

Additional file 4: Short tandem repeat (STR) profiling of 8591. (PDF 2185 kb)

Additional file 5: **Figure S1.** Flow cytometry (FCM) analyses of LGR5 in glioma cells. (**a**) FCM analyses of LGR5 positive proportion in parent and enriched cells. (**b**) Fluorescence-activated cell sorting (FACS) of LGR5^+^ cells in U251 glioma cells and 8591 primary glioma cells. (PPTX 2348 kb)

Additional file 6: **Figure S2.** Stemness properties of LGR5^+^ 8591 cells in vitro. (**a**) Cell proliferation assays of LGR5^+^ and LGR5^−^ 8591 cells (*P* < 0.001, *n* = 3, two-way ANOVA). (**b**) Coloning sphere images and sphere numbers in the clone formation assay (*P* < 0.001, *n* = 3, Student *t* test). Scale bar = 100 μm. (**c**) Drug resistance curve of TMZ in LGR5^+^ and LGR5^−^ 8591 cells. (**d**) Images and numbers of invasive cells in invasion assays (top, *P* < 0.001, *n* = 3, Student *t* test) and images and numbers of migrated cells in migration assays (bottom, *P* < 0.001, *n* = 3, Student *t* test). All data are represented as mean ± SD from triplicate wells. ***, *P* < 0.001, as compared to control. (PPTX 7790 kb)

Additional file 7: **Figure S3.** Stemness properties of LGR5^+^ 8591 cells in vivo. (**a**) Xenografts produced by LGR5^+^ and LGR5^−^ 8591 cells. (**b**) The subcutaneous xenografts growth rate generated by LGR5^+^ and LGR5^−^ 8591 cells in BALB/c-nu mice (two-way ANOVA, *P* = 0.001). Data are shown as the mean ± SD (LGR5^+^ 8591: *n* = 5, LGR5^−^ 8591: *n* = 5). (**c**) IHC staining of LGR5, Ki67, N-cadherin, SOX2and CD44 expression in LGR5^+^ xenografts and LGR5^−^ xenografts (magnification × 200). (**d**) The percentage of positive expression of LGR5, Ki67, N-cadherin, SOX2and CD44 in LGR5^+^ xenografts and LGR5^−^ xenografts (*n* = 5, Student *t* test). Error bars represent the mean ± SD. (**e**) The correlation between the levels of LGR5 and Ki67 by Spearman correlation analysis (*P* < 0.05, *r* = 0.7505, *n* = 10). (**f**) The correlation between the levels of LGR5 and N-cadherin by Spearman correlation analysis (*P* < 0.001, *r* = 0.8989, *n* = 10). (**g**) The correlation between the levels of LGR5 and SOX2 by Spearman correlation analysis (*P* < 0.01, *r* = 0.7939, *n* = 10). (**h**) The correlation between the levels of LGR5 and CD44 by Spearman correlation analysis (*P* < 0.01, *r* = 0.8182, *n* = 10). (**i**) Double-staining of LGR5 and N-cadherin in 8591 subcutaneous xenografts. The xenografts edge (section “3 and 4”) showed many positive cells expressed both LGR5 and N-cadherin, while there was few positive cells in inside xenografts (section “Methods”). Scale bar = 100 μm. *, *P* < 0.05; **, *P* < 0.01; ***, *P* < 0.001. (PPTX 6203 kb)

Additional file 8: **Figure S4.** The transfection efficiency of transfected GSCs. (**a**) The transfection efficiency of transfected U251 GSCs by FCM analyses. (**b**) The transfection efficiency of transfected U251 GSCs by FCM analyses. (PPTX 2671 kb)

Additional file 9: **Figure S5.** The representative T2W images of peritumoral edema in the HGG group and the LGG group. The degree of peritumoral edema was divided into two groups according to the following criteria: maximum diameter > 2 cm, severe; maximum diameter ≦ 2 cm, slight/None. (PPTX 1049 kb)

Additional file 10: **Table S3.** Univariate analysis of factors associated with prognosis in the HGG group. (XLS 13 kb)

Additional file 11: **Table S4.** Univariate analysis of factors associated with prognosis in the LGG group. (XLS 12 kb)

## Abbreviations

CSCs: Cancer stem cells
EMT: Epithelial-mesenchymal transition
EpCAM: Epithelial cell adhesion molecule
FACS: Fluorescence-activated cell sorting
FCM: Flow cytometry
GBM: Glioblastoma multiforme
GFAP: Glial fibrillary acidic protein
GSCs: Glioma stem cells
HGG: High-grade glioma
IDH1: Isocitrate dehydrogenase 1
IF: Immunofluorescence
IHC: Immunohistochemistry
KPS: Karnofsky performance scale
LGG: Low-grade glioma
LGR5: Leucine-rich repeat-containing G protein-coupled receptor 5
OS: Overall survival
PFS: Progression-free survival
STR: Short tandem repeat
TMZ: Temozolomide
