# Supplementary material for: LGR5, a novel functional glioma stem cell marker, promotes EMT by activating the Wnt/β-catenin pathway and predicts poor survival of glioma patients
Source: J Exp Clin Cancer Res. 2018 Sep 12;37:225. doi: 10.1186/s13046-018-0864-6 (PMC6136228; doi:10.1186/s13046-018-0864-6)

GENETIC TESTING BIOTECHNOLOGY

Cell Line Authentication Service

STR Profile Report

Sample Submitted By: Dr. Jinghai Wan, Dr. Jin Zhang
Cancer Hospital, National Cancer Centre of China, Chinese Academy of Medical Sciences

Email Address: wanjinghai@sina.com; mydear07@163.com

Sales Order: 171121A

Cell Line Designation: U251

Date Sample Received: Nov 21th, 2017

Report Date: Nov 22th, 2017

Methodology: Nineteen short tandem repeat (STR) loci plus the gender determining locus, Amelogenin, were amplified using the commercially available EX20 Kit from AGCU. The cell line sample was processed using the ABI Prism® 3500 Genetic Analyzer. Data were analyzed using GeneMapper® ID-X v1.4 software (Applied Biosystems). Appropriate positive and negative controls were run and confirmed for each sample submitted.

Data Interpretation: Cell lines were authenticated using Short Tandem Repeat (STR) analysis as described in 2012 in ANSI Standard (ASN-0002) by the ATCC Standards Development Organization (SDO) and in Capes-Davis et al., Match criteria for human cell line authentication: Where do we draw the line? Int J Cancer. 2013;132(11):2510-9.

GTB™ performs STR Profiling following ISO 9001:2008 and ISO/IEC 17025:2005 quality standards.
There are no warranties with respect to the services or results supplied, express or implied, including, without limitation, any implied warranty of merchantability or fitness for a particular purpose. Genetic Testing Biotechnology (GTB) is not liable for any damages or injuries resulting from receipt and/or improper, inappropriate, negligent or other wrongful use of the test results supplied, and/or from misidentification, misrepresentation, or lack of accuracy of those results. Your exclusive remedy against GTB and those supplying materials used in the services for any losses or damage of any kind whatsoever, whether in contract, tort, or otherwise, shall be, at GTB's option, refund of the fee paid for such service or repeat of the service.

The GTB™ is a registered trademark of Genetic Testing Biotechnology Corporation (Suzhou).

Technical Questions?
GTB Technical Support
+86-512-62806339
STR_service@163.com
Section 303, Yixin BLD
SIP, Suzhou, 215123
Jiangsu, P.R. China

Ordering Questions?
STR_order@163.com
GTB Corporation
+86-512-62806339
Section 303, Yixin BLD
SIP, Suzhou, 215123
Jiangsu, P.R. China

GENETIC TESTING BIOTECHNOLOGY

Cell Line Authentication Service

STR Profile Report

Sales Order: 171121A

Test Results for Submitted Sample			DSMZ Reference Database Profile	
**Loci**	**Query Profile:** U251		**Database Profile:** U251 MG	
Amelogenin	X	Y	X	Y
D3S1358	16	17		
D13S317	10	11	10	11
D7S820	10	12	10	12
D16S539	12		12	
Penta E	7	10		
TPOX	8		8	
TH01	9.3		9.3	
D2S1338	22	24		
CSF1PO	11	12	11	12
Penta D	12			
D19S433	13	15		
vWA	16	18	16	18
D21S11	29			
D18S51	13			
D6S1043	12			
D8S1179	13	15		
D5S818	11	12	11	12
D12S391	17	22		
FGA	21	25		

The allele match algorithm compares the 8 core loci plus amelogenin only, even though alleles from all loci will be reported when available.

Note: Loci highlighted in grey (8 core STR loci plus Amelogenin) can be made public to verify cell identity. In order to protect the identity of the donor, ***please do not publish*** *the allele calls from all the STR loci tested.*

Explanation of Test Results

Cell lines with ≥80% match are considered to be related; i.e., derived from a common ancestry. Cell lines with between a 55% to 80% match require further profiling for authentication of relatedness.

- ☐ The submitted sample profile is human, but not a match for any profile in the DSMZ STR database.
- ☑ The submitted profile is an exact match for the following human cell line(s) in the DSMZ STR database (8 core loci plus Amelogenin): U251 MG
- ☐ The submitted profile is similar to the following DSMZ human cell line(s):

e-Signature, Technician:

Digitally signed by Faye Wong
DN: cn=Faye Wong, o=Genetic Testing Biotechnology (Suzhou), ou=DNA Typing Section, email=str_service@163.com, c=CN
Date: 2017.11.22 16:31:12 +08'00'

GENETIC TESTING BIOTECHNOLOGY – GUARANTEE – CELL LINE AUTHENTICATION

e-Signature, Reviewer:

Digitally signed by Alan Cui
DN: cn=Alan Cui, o=Genetic Testing Biotechnology Corporation (Suzhou), ou=Supervision Section, email=str_order@163.com, c=CN
Date: 2017.11.22 16:32:18 +08'00'

More information

Addendum: Electropherogram/matching results for the customer's sample set 1 of 1

GENETIC TESTING BIOTECHNOLOGY

Cell Line Authentication Service

STR Profile Report

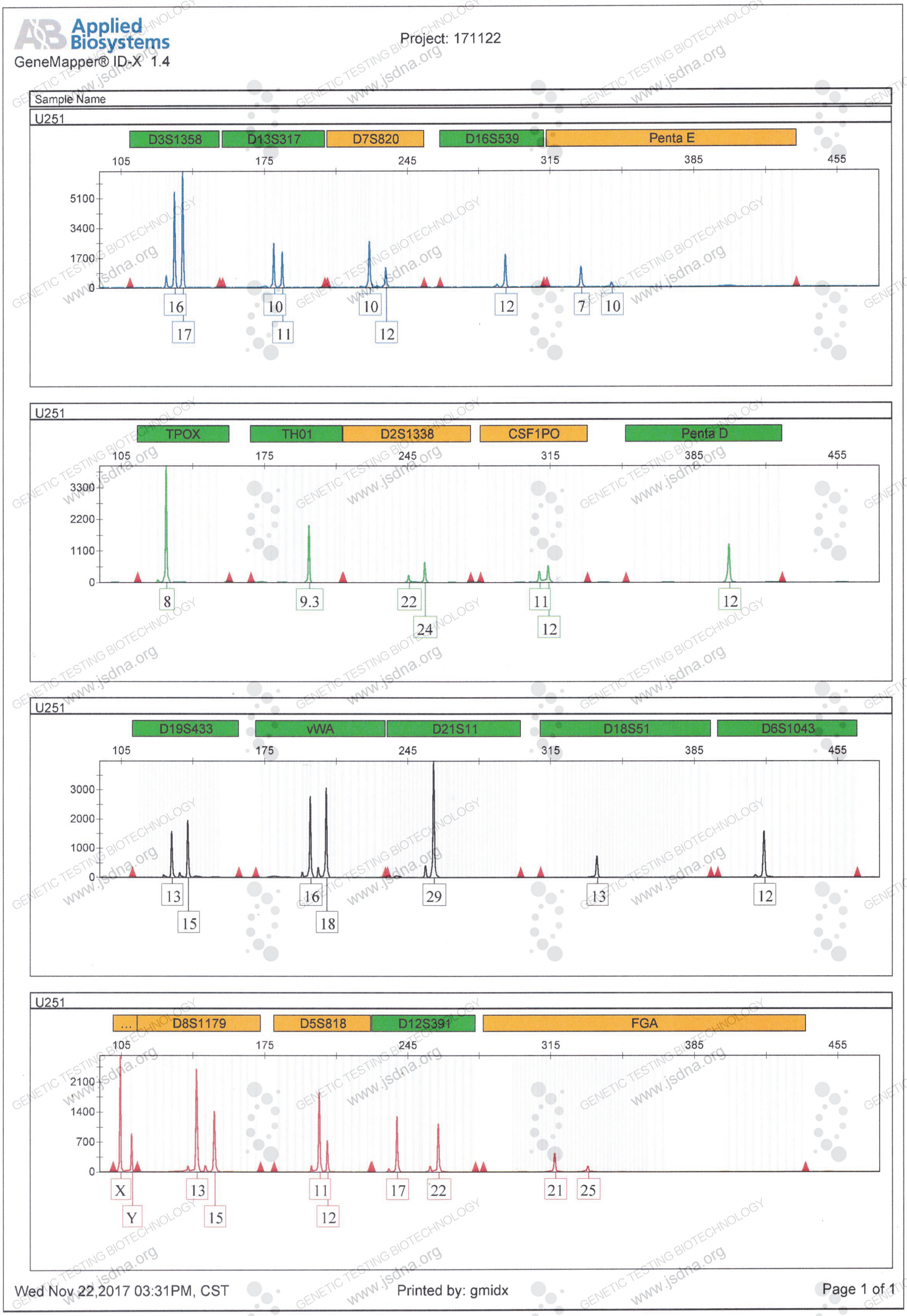

Supplement: Supplementary file 3 — Short tandem repeat (STR) profiling of U251. (PDF 2190 kb) [file 13046_2018_864_MOESM3_ESM.pdf]
