# Supplementary figures and images for: LGR5, a novel functional glioma stem cell marker, promotes EMT by activating the Wnt/β-catenin pathway and predicts poor survival of glioma patients
Source: J Exp Clin Cancer Res. 2018 Sep 12;37:225. doi: 10.1186/s13046-018-0864-6 (PMC6136228; doi:10.1186/s13046-018-0864-6)

## Slide 1
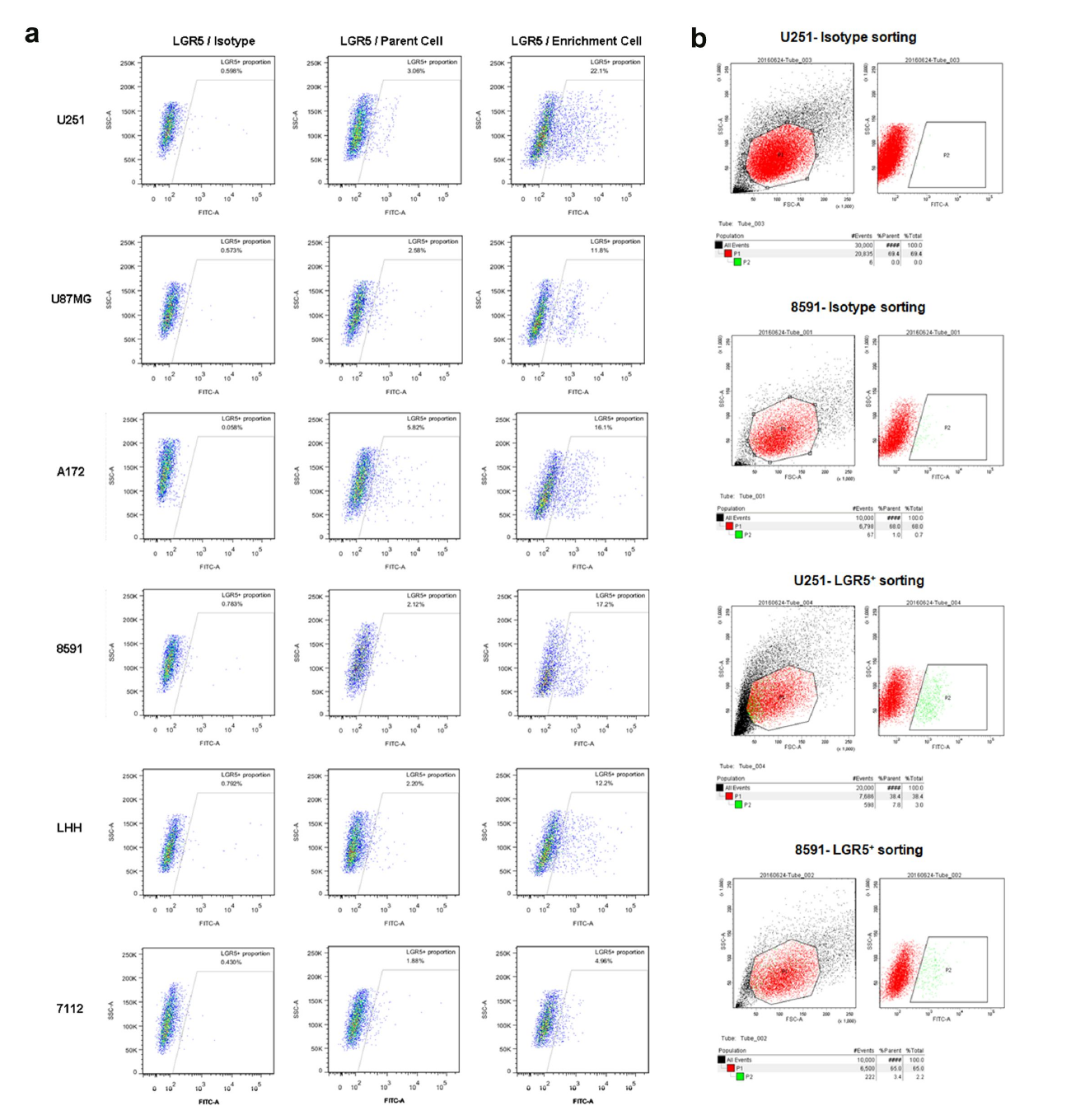

Supplement: Supplementary file 5 — Figure S1. Flow cytometry (FCM) analyses of LGR5 in glioma cells. (a) FCM analyses of LGR5 positive proportion in parent and enriched cells. (b) Fluorescence-activated cell sorting (FACS) of LGR5+ cells in U251 glioma cells and 8591 primary glioma cells. (PPTX 2348 kb) [file 13046_2018_864_MOESM5_ESM.pptx]

## Slide 1
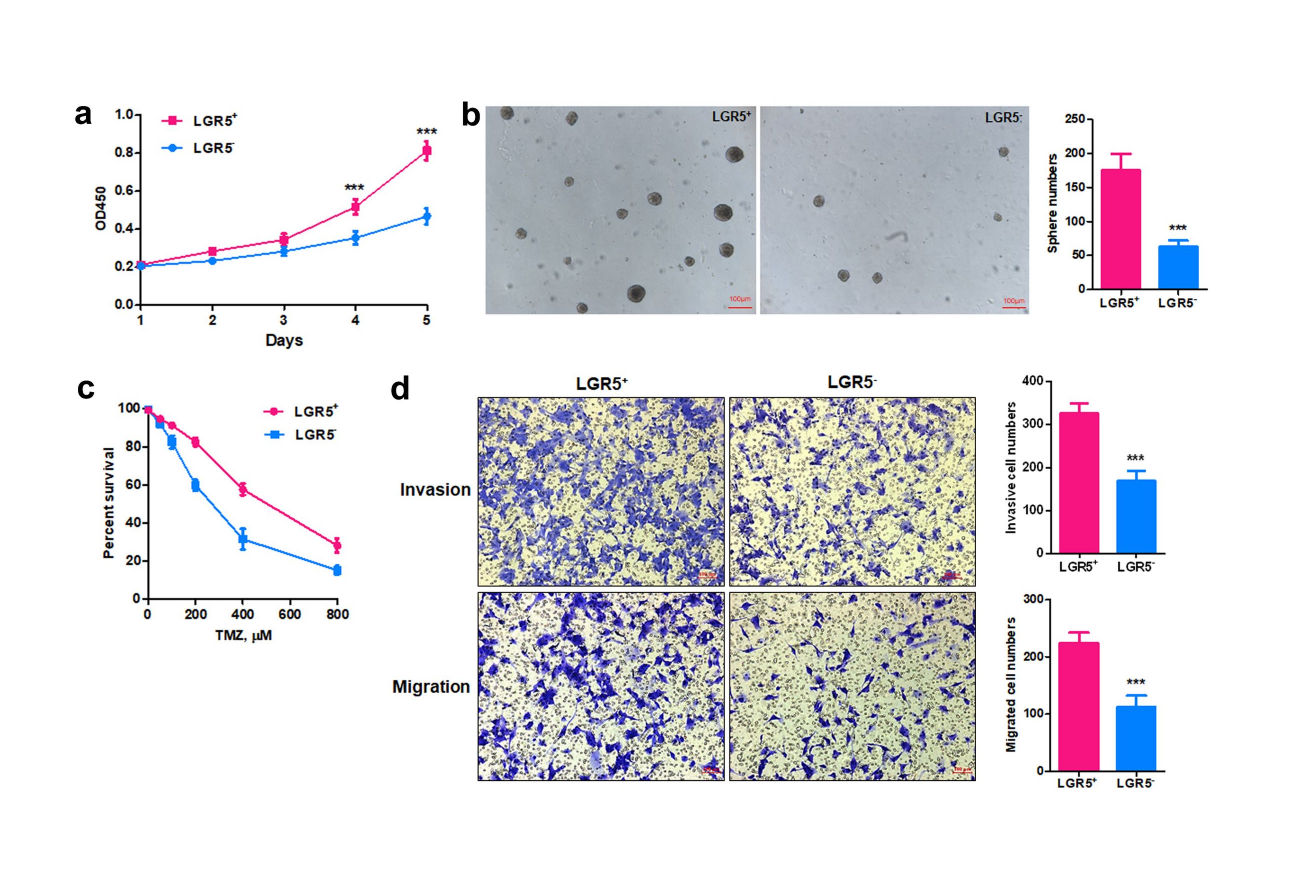

Supplement: Supplementary file 6 — Figure S2. Stemness properties of LGR5+ 8591 cells in vitro. (a) Cell proliferation assays of LGR5+ and LGR5− 8591 cells (P < 0.001, n = 3, two-way ANOVA). (b) Coloning sphere images and sphere numbers in the clone formation assay (P < 0.001, n = 3, Student t test). Scale bar = 100 μm. (c) Drug resistance curve of TMZ in LGR5+ and LGR5− 8591 cells. (d) Images and numbers of invasive cells in invasion assays (top, P < 0.001, n = 3, Student t test) and images and numbers of migrated cells in migration assays (bottom, P < 0.001, n = 3, Student t test). All data are represented as mean ± SD from triplicate wells. ***, P < 0.001, as compared to control. (PPTX 7790 kb) [file 13046_2018_864_MOESM6_ESM.pptx]

## Slide 1
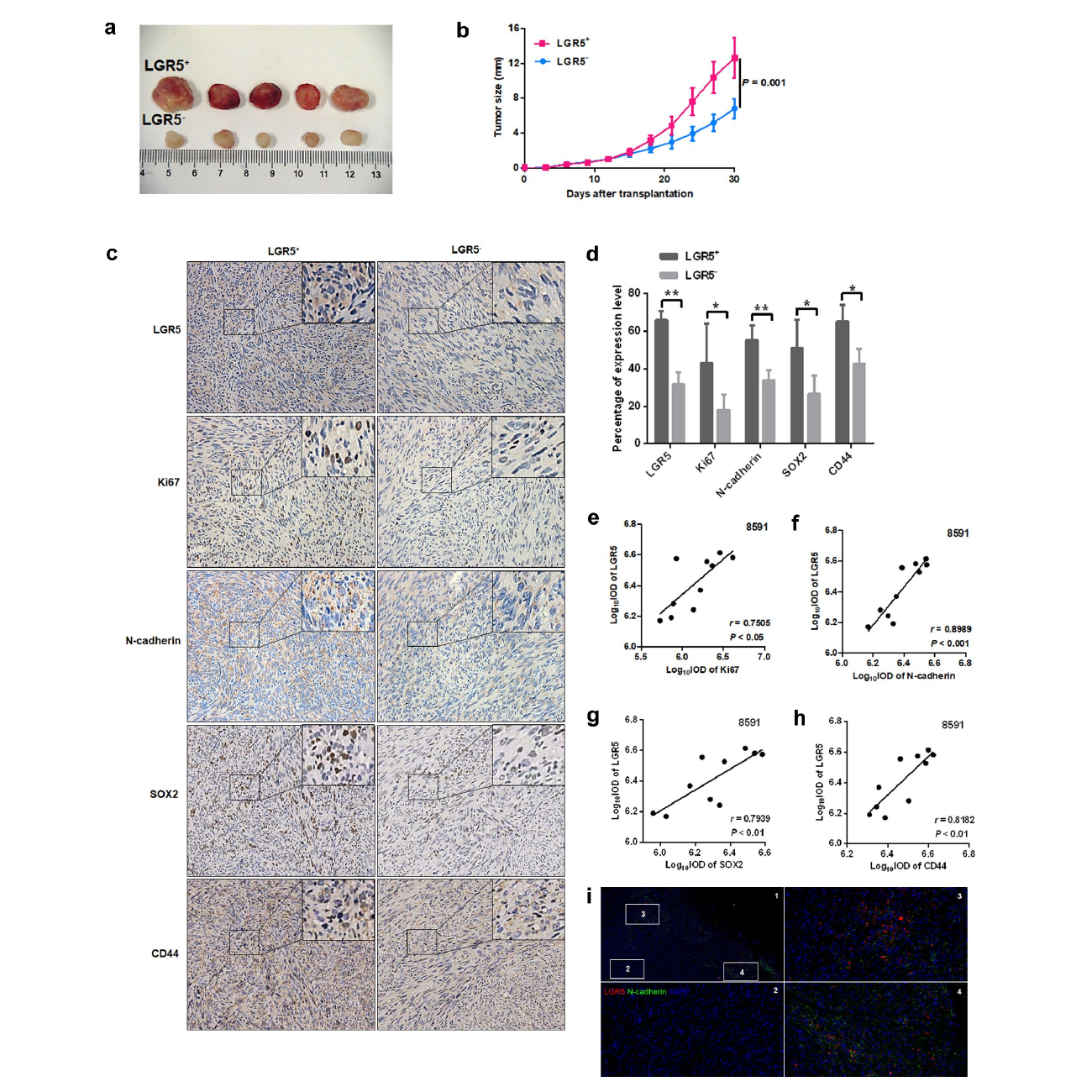

Supplement: Supplementary file 7 — Figure S3. Stemness properties of LGR5+ 8591 cells in vivo. (a) Xenografts produced by LGR5+ and LGR5− 8591 cells. (b) The subcutaneous xenografts growth rate generated by LGR5+ and LGR5− 8591 cells in BALB/c-nu mice (two-way ANOVA, P = 0.001). Data are shown as the mean ± SD (LGR5+ 8591: n = 5, LGR5− 8591: n = 5). (c) IHC staining of LGR5, Ki67, N-cadherin, SOX2and CD44 expression in LGR5+ xenografts and LGR5− xenografts (magnification × 200). (d) The percentage of positive expression of LGR5, Ki67, N-cadherin, SOX2and CD44 in LGR5+ xenografts and LGR5− xenografts (n = 5, Student t test). Error bars represent the mean ± SD. (e) The correlation between the levels of LGR5 and Ki67 by Spearman correlation analysis (P < 0.05, r = 0.7505, n = 10). (f) The correlation between the levels of LGR5 and N-cadherin by Spearman correlation analysis (P < 0.001, r = 0.8989, n = 10). (g) The correlation between the levels of LGR5 and SOX2 by Spearman correlation analysis (P < 0.01, r = 0.7939, n = 10). (h) The correlation between the levels of LGR5 and CD44 by Spearman correlation analysis (P < 0.01, r = 0.8182, n = 10). (i) Double-staining of LGR5 and N-cadherin in 8591 subcutaneous xenografts. The xenografts edge (section “3 and 4”) showed many positive cells expressed both LGR5 and N-cadherin, while there was few positive cells in inside xenografts (section “Methods”). Scale bar = 100 μm. *, P < 0.05; **, P < 0.01; ***, P < 0.001. (PPTX 6203 kb) [file 13046_2018_864_MOESM7_ESM.pptx]

## Slide 1
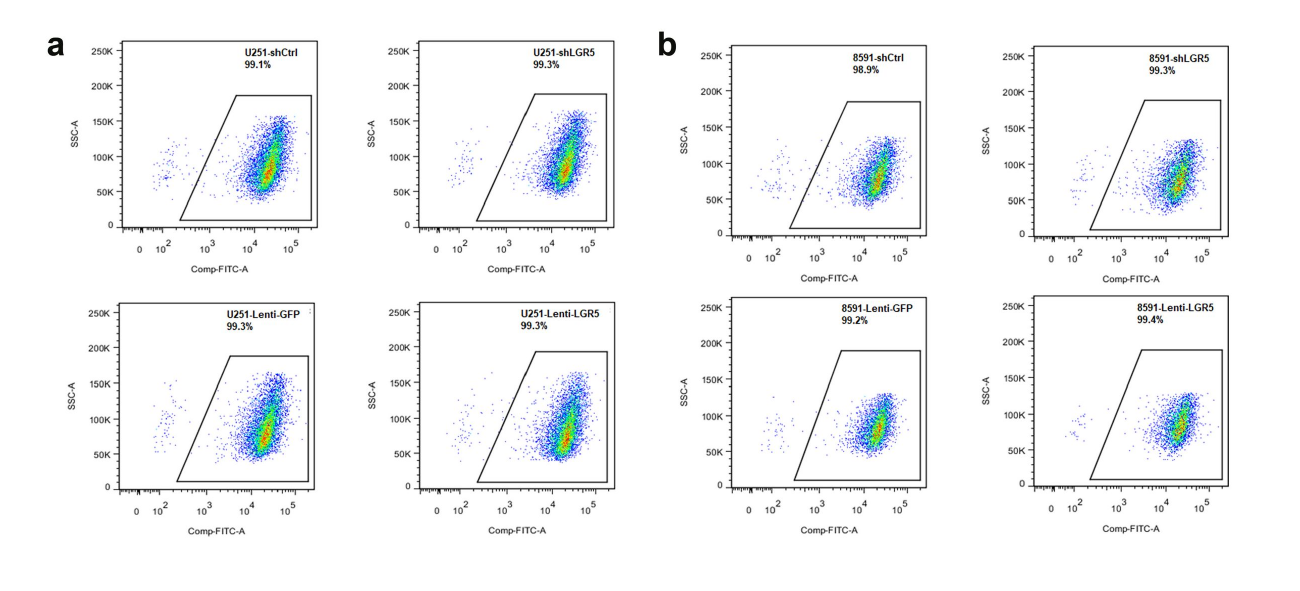

Supplement: Supplementary file 8 — Figure S4. The transfection efficiency of transfected GSCs. (a) The transfection efficiency of transfected U251 GSCs by FCM analyses. (b) The transfection efficiency of transfected U251 GSCs by FCM analyses. (PPTX 2671 kb) [file 13046_2018_864_MOESM8_ESM.pptx]

## Slide 1
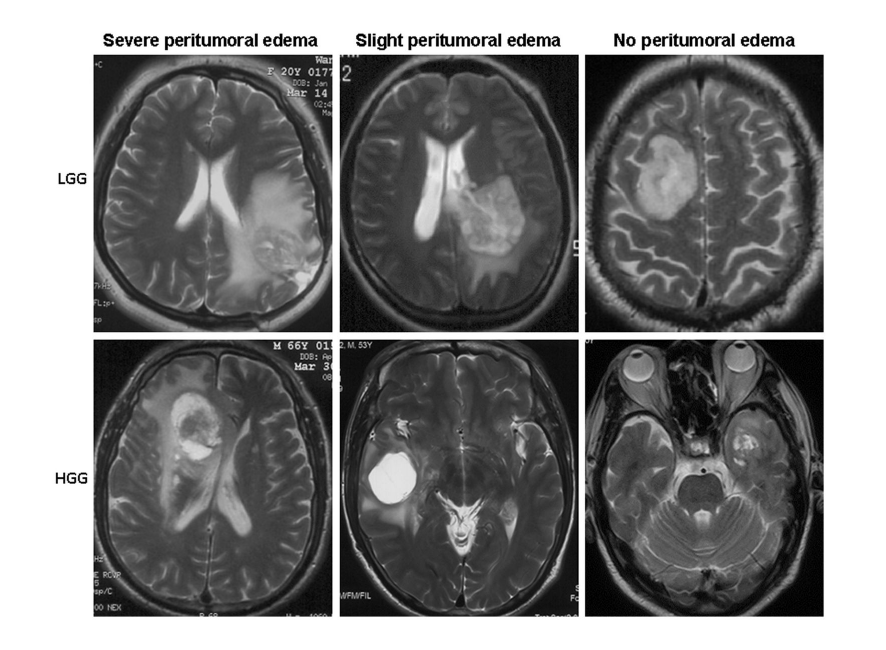

Supplement: Supplementary file 9 — Figure S5. The representative T2W images of peritumoral edema in the HGG group and the LGG group. The degree of peritumoral edema was divided into two groups according to the following criteria: maximum diameter > 2 cm, severe; maximum diameter ≦ 2 cm, slight/None. (PPTX 1049 kb) [file 13046_2018_864_MOESM9_ESM.pptx]
